# Insights to the HIV-associated visceral leishmaniasis clinical outcome: lessons learned about immune mediated disorders

**DOI:** 10.3389/fimmu.2025.1516176

**Published:** 2025-03-12

**Authors:** Maria Luciana Silva-Freitas, Gabriela Corrêa-Castro, Alda Maria Da-Cruz, Joanna Reis Santos-Oliveira

**Affiliations:** ^1^ Laboratório Interdisciplinar de Pesquisas Médicas - Instituto Oswaldo Cruz – Fundação Oswaldo Cruz (FIOCRUZ), Rio de Janeiro, Brazil; ^2^ Departamento de Microbiologia, Immunologia e Parasitologia (DMIP), Faculdade de Ciências Médicas, Universidade do Estado do Rio de Janeiro (UERJ), Rio de Janeiro, Brazil; ^3^ Instituto Nacional de Ciência, Tecnologia e Inovação - Neuroimunomodulação (INCT - NIM), Conselho Nacional de Desenvolvimento Científico e Tecnológico (CNPq), Brasília, Brazil; ^4^ Departamento de Doenças Transmissíveis, Secretaria de Vigilância em Saúde e Ambiente, Ministério da Saúde, Brasília, Brazil; ^5^ Núcleo de Ciências Biomédicas Aplicadas, Instituto Federal de Educação, Ciência e Tecnologia do Rio de Janeiro (IFRJ), Rio de Janeiro, Brazil

**Keywords:** VL/HIV co-infection, visceral leishmaniasis, immune response, cellular activation, immunosenescence, relapses

## Abstract

Most cases of visceral leishmaniasis (VL) and human immunodeficiency virus (HIV) co-infection (VL/HIV) in the Americas occur in Brazil, and the prevalence of VL/HIV has been increasing since 2019, reaching 19% in 2023. This association presents a challenge for the management of VL, since both VL and HIV infection share immunopathogenic characteristics that can reciprocally affect co-infected patients. Thus, VL may contribute to the immunosuppression and other immunological disturbances associated with the rapid progression to acquired immunodeficiency syndrome (AIDS), whereas HIV infection accelerates the development of active VL and reduces the probability of a successful response to anti-*Leishmania* therapy, resulting in an increase in the relapse and lethality rates of VL. In this synergistic impairment, one of the most critical hallmarks of VL/HIV co-infection is the enhancement of immunosuppression and intense chronic immune activation, caused not only by each infection per se, but also by the cytokine storm and translocation of microbial products. Thus, co-infected patients present with an impaired effector immune response that may result in inefficient parasitic control. In addition, the chronic activation environment in VL/HIV patients may favor progression to early immunosenescence and exhaustion, worsening the patients’ clinical condition and increasing the frequency of disease relapse. Herein, we review the immunological parameters associated with the immunopathogenesis of VL/HIV co-infection that could serve as good biomarkers of clinical prognosis in terms of relapse and severity of VL.

## Introduction

The human immunodeficiency virus (HIV)/acquired immunodeficiency syndrome (AIDS) pandemic over the last 40 years has modified the clinical and epidemiological spectrum of leishmaniasis. An overlap between the areas of visceral leishmaniasis (VL) transmission and HIV infection has been clearly observed, with one-third of HIV cases worldwide occurring in areas at risk of leishmaniasis transmission (1, 2). Consequently, co-infection of VL with HIV (VL/HIV) has emerged as an important challenge in VL control, with VL itself becoming an important opportunistic disease associated with HIV infection (3). Herein, we carefully explain the main immunopathogenic mechanisms underlying the association between visceral leishmaniasis and HIV infection. Moreover, we also pointed out how the knowledge generated from cross-sectional and longitudinal studies helped to predict the different clinical outcomes of these patients, particularly in terms of disease severity or relapses.

## Epidemiology of VL/HIV co-infection

Since the 1980s, when the first case of leishmaniasis associated with HIV infection was published (4), an increase in the cases of co-infection has been recorded. In the 1990s, the majority of co-infection cases were reported in European countries in the Mediterranean region (Spain, France, Italy, and Portugal), where 1.5% to 9% of individuals with AIDS presented with new cases or reactivation of infection by viscerotropic species of *Leishmania* (5). The prevalence of VL in this population was 500 times higher than that in the non-HIV-infected population (6).

Subsequently, the advent of combined antiretroviral therapy (cART) modified this epidemiological scenario, resulting in a progressive decrease in VL/HIV cases in the Mediterranean basin and a considerably low incidence in this region (7). Nevertheless, 45 countries worldwide have reported cases of *Leishmania*/HIV co-infection, with the visceral form being the most prevalent (3). The most critical incidence scenario has been reported in some African countries, such as Sudan and Ethiopia, where 35% of individuals with VL have HIV co-infection, as well as in the state of Bihar in India and the countries in Central and South America, particularly Brazil.

Considering the elevated number of cases of both infections in Brazil, it is also expected to have the highest incidence of VL/HIV co-infection in the American continent. This epidemiological profile can be attributed to the spread of HIV infection to rural areas, urbanization of the VL vector, or even the evident urban problem of sharing of contaminated needles by drug users (8). In addition, some factors related to diagnosis may also be involved in this highest incidence. Firstly, the greater predisposition to symptomatic VL between immunosuppressed by HIV infection can lead to the opening of a VL case, or even to the reactivation of a latent infection. Moreover, the occurrence of VL/HIV in urban areas often facilitates the search for medical consultations and a faster parasitological diagnosis. Finally, it is believed that the computerized health notification system, together with the existence of databases that allow cross-referencing between them, can allow for better monitoring and notification by surveillance system. In this way, since 2019, the prevalence of VL/HIV co-infection has progressively increased, reaching >11% (9), which may still be underreported due to a reduction in the number of confirmed cases of VL during the coronavirus disease 2019 pandemic. Therefore, although the prevalence has increased, it is important to consider that these percentages only reflect cases showing clinical manifestations of VL; asymptomatic cases may be diagnosed late, and a significant number of patients with VL do not undergo serological investigation for HIV (10), indicating that the actual scenario is even more worrying.

In 2023, approximately 300 new cases of VL/HIV co-infection were reported to the Brazilian Ministry of Health, accounting for approximately 19% of the VL cases reported during this period (9). In line with the simultaneous expansion and geographic overlap of both infections, most cases of VL/HIV co-infection in Brazil have been reported in the Northeast, Midwest, and Southeast regions, especially in the states of Maranhão, Mato Grosso do Sul, and Minas Gerais, respectively (9). Young adult males aged 20-49 years and injection drug users are the most affected groups, representing an ever-expanding exposure category (11). Additionally, in 41% of the cases, the diagnosis of both infections occurred simultaneously, with VL being responsible for opening up an HIV/AIDS case (11). In contrast, latent VL has also been reported in HIV-infected individuals, who show a higher risk of VL relapse, especially when CD4^+^ T-lymphocyte absolute counts reach levels < 200 cells/mm^3^, making them possible reservoirs for the parasite (12).

Then, one important factor is that *Leishmania* and HIV infection reinforce each other, posing significant clinical and public health problems. It is worth noting that important socio-demographic issues also permeate this association at Brazil. Social vulnerability places VL/HIV patients in conditions of malnutrition and exposure to factors that may favor the development of symptomatic VL. On the other hand, patients who commonly come from rural areas experience geographical limitations and mobility difficulties that can make difficult their access to effective treatment and clinical monitoring offered at reference centers in urban areas. Therefore, providing strategies that go beyond the currently recommended protocols, particularly with regard to predicting prognosis, can contribute to long-term clinical improvement, resulting in quality of life for the patient and lower costs for the healthcare system.

## Evidence for VL as an HIV/AIDS-related disease

Although it is not yet considered an AIDS-defining disease, VL may contribute to the immunosuppression and other immunological disturbances associated with rapid progression to AIDS (13–16). However, HIV co-infection has been shown to accelerate the development of active VL and reduce the probability of a successful response to leishmanial therapy, increasing the VL lethality rate and enhancing the predisposition to VL relapse by 3-5 times in comparison with that in HIV-negative individuals (10, 12, 17). In this context, *Leishmania/*HIV co-infection represents a major challenge for management of VL, since both infections share immunopathogenic characteristics that can reciprocally affect co-infected patients.

**Figure 1:**
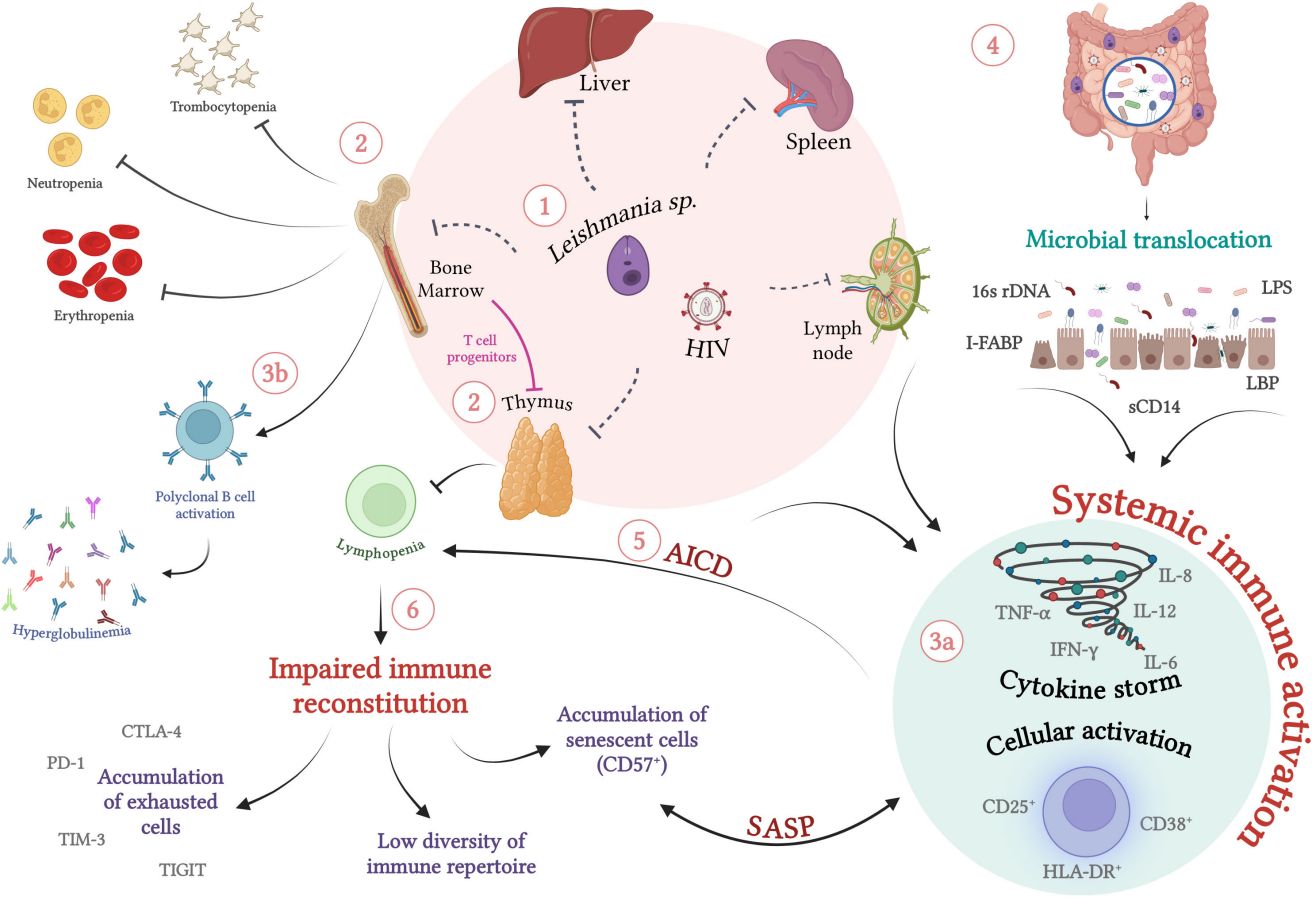
Immunopathogenesis of visceral leishmaniasis in HIV-infected individuals: Chronic immune activation and its implications for the immune impairment of VL/HIV co-infection (1) Co-infection with *L. infantum*/*L. donovani* and HIV is characterized by simultaneous systemic involvement of organs such as lymph nodes and thymus, in addition to parasitism of the bone marrow, spleen and liver (2). The intense parasitism of monocytes and macrophages by the protozoan culminates in changes in the production of myeloid and lymphoid progenitors by the bone marrow. In an attempt to recover the parasitized cells, there is a shift in favor of this lineage, which can lead to a laboratorial profile of erythropenia, neutropenia, thrombocytopenia and lymphopenia. In VL/HIV co-infection, the drop in absolute counts of peripheral CD4 T lymphocytes is enhanced by infection and concomitant thymic impairment by HIV and the protozoan. (3a) In parallel with immunosuppression, co-infection occurs with high cellular activation, characterized by activated T lymphocytes, high production of inflammatory cytokines (cytokine storm) and (3b) polyclonal activation of B lymphocytes, which culminates in hyperglobulinemia (4). In addition to parasite and viral antigens, the translocation of microbial products has been identified as an important cofactor for increasing levels of systemic activation (5). As a result of this persistent activation, activation-induced cell death (AICD), worsening of lymphopenia and an impairment of the cellular immune response specific to the parasite is observed, which may favor relapses (6). The maintenance of this scenario can result in the exhaustion of primary immune resources (thymus and bone marrow) and the inability of lymphocytic repopulation. This process results in the deterioration of the responsive capacity to parasitic stimuli, favoring the accumulation of exhausted and/or terminally differentiated cells and the low diversity of the lymphocyte repertoire. SASP, Senescence-Associated Secretory Phenotype. Dashed lines indicate direct damage caused by pathogens. Source: The author. Created with BioRender.com.

VL immunopathogenesis is characterized by systemic involvement, since the amastigote forms of *L. (L.) infantum* and *L. (L.) donovani* show marked tropism for mononuclear phagocytic cells of the spleen, liver, bone marrow, and lymph nodes. This leads to impaired host immune defense mechanisms because of a shift in the production of myeloid and lymphoid cells in the bone marrow as a result of intense parasitism and destruction of mononuclear cells and the consequent attempts to replace them. In laboratory assessments, classic pancytopenia is characterized mainly by neutropenia, lymphopenia, anemia, and thrombocytopenia (18–21). Lymphopenia may be caused by deviations in the production of T-lymphocyte progenitors and by thymic atrophy as a result of undernutrition and parasitism (22–24). Additionally, activation-induced cell death in the periphery may be associated with lymphopenia. More recently, CD4^+^ T-cell depletion has been suggested to be caused by pathogenic changes in the spleen due to advanced white pulp disorganization and splenic depletion of CD4^+^ T cells by apoptosis and pyroptosis secondary to HIV and parasite infection (25). Independent of the mechanisms involved, all of them can contribute to the impairment of a specific immune response against the parasite, characterizing VL as an immunosuppressive disease (**Figure 1**).

Indeed, the evolution of active VL is characterized by specific immunosuppression for parasite antigens since the delayed-type hypersensitivity test (Montenegro test) shows negative results, unlike in individuals with asymptomatic or subclinical disease (26–28). In addition, patients with active VL show a decrease in the proliferative capacity of helper T cells when stimulated *in vitro* with antigens of the parasite and also a decrease in the production of specific cytokines such as interleukin (IL)-2 and interferon (IFN)-γ, alongside an increase of IL-10 (26, 27, 29, 30).

Absolute CD4^+^ T-cell counts play a key role in the prognosis of VL in terms of immunosuppression and immunological reconstitution. Patients with VL show lower CD4^+^ T-cell counts than healthy individuals. Although the CD4^+^ T-cell counts recover during the remission phase, they do not reach normal levels (31). A recent study of our group showed that this CD4^+^ T-cell depletion is associated with the clinical prognosis. Patients with non-relapsed VL showed a significant increase in the number of these cells shortly after treatment, which was not observed in patients with relapse (32). Although this evidence indicates the importance of evaluating this parameter in clinical practice, it is not routinely assessed in patients with non-HIV VL.

Paradoxically, VL immunopathogenesis evolves with an intense degree of activation of the immune system. Our group demonstrated that patients with active VL present high percentages of activated T lymphocytes, which remain elevated even after clinical remission (31). However, when these cells were stimulated with *Leishmania* antigens, the percentage of activated cells was lower than that of lymphocytes from individuals in clinical remission, corroborating the specific immunosuppressive profile of the active phase of VL (33). Additionally, other studies have suggested that VL is characterized by an exacerbated systemic inflammatory response mediated by inflammatory cytokines such as IL-8, tumor necrosis factor (TNF), and IL-6, especially in active disease (34, 35). This inflammatory and activated status has been associated with multiple organ failure, showing similarities with the findings observed in sepsis, malaria, and other inflammatory diseases (36, 37).

Similar immunopathogenic aspects have been observed in patients with HIV infection. CD4+ T lymphocytes play a central role in the acquired immune responses against HIV. As the infection progresses, the continuous loss of these cells, especially to levels below 200 cells/mm^3^, favors opportunistic infections such as tuberculosis or VL (38, 39).

The persistence of the anti-HIV immune response, in addition to factors that go beyond viral antigens, leads to chronic immune activation (40–42). High levels of pro-inflammatory cytokines, such as TNF, IL-6, and IL-1β, and chemokines, such as regulated on activation, normal T-cell expressed and secreted (RANTES), macrophage inflammatory protein (MIP)-1α, MIP-1β, and chemokine ligand 13 (CXCL13) have also been directly implicated in this process (43–47).

Since *Leishmania* sp. and HIV infect the same target cells and thus compromise the same immune compartments, they can be reasonably expected to cause reciprocal impairment of the effector immune response and, consequently, of the control of each pathogen (48). The immunological alterations observed in VL and HIV/AIDS can synergistically affect co-infected patients. Therefore, one of the most critical hallmarks of VL/HIV co-infection is severe immunosuppression and intense chronic immune activation (**Figure 1**), especially that caused by a cytokine storm, such that VL results in worsening of the clinical condition of patients co-infected with HIV.

Indeed, the chronic stimulation observed in VL can increase viral replication and latent provirus expression, resulting in faster progression to AIDS (49). *Leishmania* spp. can augment viral replication by inducing cellular activation and an inflammatory microenvironment, which in turn increases the susceptibility of target cells to infection (50–52). Indeed, the lipophosphoglycan (LPG) molecule presenting on the parasite’s surface activates the transcription factor nuclear factor (NF)-ƙB, increasing TNF production (53, 54). However, the role of LPG in the *Leishmania*-HIV interaction is still controversial because LPG also inhibits viral entry into monocyte-derived macrophages (MDMs) in the early phase of infection, resulting in reduced viral replication (55).

*L. infantum*-infected human dendritic cells co-cultured with autologous CD4^+^ T lymphocytes show increased viral replication, probably through the secretion of cytokines such as IL-6 and TNF (51). Accordingly, VL/HIV co-infected patients present with high levels of TNF and IL-6, even with the use of cART and anti-*Leishmania* treatment, which may be associated with high viremia and progressive loss of CD4^+^ T lymphocytes (56–58).

In addition to influencing the viral load of patients co-infected with HIV, VL can potentiate the depletion of CD4^+^ T lymphocytes (59). Low absolute counts of CD4^+^ T lymphocytes have been observed in VL/HIV-co-infected patients during the VL active phase and clinical remission despite cART (60, 61). Notably, these counts were lower than those observed in individuals with HIV mono-infection (61). Takele et al. also reported low counts of CD4^+^ T cells in the active phase in co-infected patients and suggested that these low counts may be associated with low production of antigen-specific IFN-γ in this phase (62), further confirming that such impairments can predispose individuals to disease recurrence. Thus, VL and its immunopathogenic consequences can aggravate the immunodeficiency caused by HIV, not only enhancing the degree of immune activation in VL/HIV co-infected patients (61), but also worsening CD4^+^ T-cell depletion.

Simultaneously, the HIV-induced disorganization of the immune system and the depletion of the pool of specific T lymphocytes severely compromise the mechanisms of parasite control, contributing to a higher parasite load and progression of VL. Indeed, HIV-infected patients, especially those with CD4^+^ T-lymphocyte counts < 200 cells/mm^3^, are at a high risk of progression to symptomatic VL (12, 15, 58, 60, 63, 64). In this scenario, VL/HIV patients are more susceptible to the development of unusual clinical manifestations and/or dissemination of VL to atypical sites (skin, gastrointestinal tract, adrenal glands, cerebrospinal fluid, respiratory tract, cardiac and renal tissues, etc.) (65–67).

Furthermore, although clinical remission of VL is commonly achieved after anti-*Leishmania* treatment, parasitemia appears to persist, even if intermittently (50). This leads to a condition called “chronically active VL,” which is associated with immunological impairment and may explain the higher susceptibility of patients with VL/HIV co-infection to multiple VL relapses (12). Additionally, the greater treatment resistance and more frequent occurrence of therapeutic failure in these patients may be related to parasitic persistence after specific therapies (49). Thus, although both infections share similar immunological characteristics, co-infected patients may not present the same clinical and laboratory profiles as HIV or *Leishmania*-infected individuals.

## Post-cART immune reconstitution in patients with VL/HIV co-infection

Although cART has undoubtedly contributed to reducing the incidence of opportunistic infections in HIV patients, it does not seem to restore the immune response in patients with VL/HIV co-infection. Indeed, antiretrovirals from the protease inhibitor (PI) class have shown direct inhibitory effects on the evolutionary forms of *L. (L.) major, L. (L.) amazonensis*, *L. (V.) braziliensis* and *L. (L.) infantum in vitro* (49, 68). Moreover, the leishmanicidal activity observed in *L. major* promastigote forms has been attributed to proteasome inhibition (69). Also, PIs appear to inhibit the replication of promastigote forms, the proliferation of amastigotes within macrophages and prevent the development of lesions in infected mice (70). Interestingly, when an *L. infantum* strain was isolated from co-infected patients undergoing cART, no inhibitory effect of PIs was observed (71). Thus, while the beneficial effects of cART on the virus and, experimentally, on the parasite are indisputable, co-infected patients present VL relapse, suggesting that immune response impairment plays a key role in the clinical prognosis.

Unlike other opportunistic diseases associated with HIV, post-cART immune reconstitution is still severely impaired in VL/HIV co-infection, as shown by low CD4^+^ T-cell counts even in patients with an undetectable HIV viral load and clinical remission after treatment of VL (49, 60, 61).

This persistent immunosuppression in patients with VL/HIV co-infection corroborates the fact that few studies have reported the occurrence of VL in association with immune reconstitution inflammatory syndrome (IRIS), which is most frequently observed in post-kala-azar dermal leishmaniasis (PKDL) (72–75), followed by tegumentary leishmaniasis and uveitis (76, 77). IRIS is characterized by a transient, but sometimes severe, local and systemic inflammatory response directed against a known condition (e.g., opportunistic pathogens or autoimmune diseases) in HIV-infected patients shortly after cART initiation due to the improvement in CD4^+^ T-cell counts (78). The primary treatment of VL in cases of concomitant HIV diagnosis preceding cART (11) should reduce the available antigenic load to stimulate the pool of specific cells post-cART reconstitution, thereby reducing the risk of IRIS, similar to the findings observed in tuberculosis/HIV co-infection (79, 80).

In the context of VL relapse, the CD4^+^ T-lymphocyte count is one of the most common parameters for predicting the clinical evolution of patients with VL/HIV co-infection (12). Through prospective follow-up, we demonstrated that co-infected patients who developed several episodes of VL showed low CD4^+^ T-lymphocyte counts for up to 12 months after treatment, while patients presenting with a single episode of VL showed a significant gain in these cells after anti-*Leishmania* treatment (15). Interestingly, both relapsed and non-relapsed groups were undergoing cART and showed viral suppression (15), suggesting that the cART does not seem to be able to prevent frequent relapses, especially the visceral form of the disease (12).

In this scenario, the maintenance of anti-*Leishmania* treatment using secondary prophylaxis could hypothetically be effective in reducing disease relapse in patients with VL/HIV co-infection (12, 81). Therefore, after specific anti-*Leishmania* therapy, these patients remained on a prophylactic regimen to avoid new active episodes of the disease. According to the Brazilian Ministry of Health, secondary prophylaxis should be administered when a patient with VL/HIV co-infection shows absolute CD4⁺ T-lymphocyte counts lower than 350 cells/mm^3^, and commonly involves administration of liposomal amphotericin B at a dose of 3–5 mg/kg every two weeks (11).

However, in most cases of VL/HIV co-infection, these specific therapeutic regimens control the parasite load in the peripheral blood within a short time period, and patients still show recurrence of active disease (82–84). A recent study conducted in a Brazilian referral hospital found that VL relapses occurred in 36.4% of the patients with VL/HIV co-infection receiving secondary prophylaxis (85). The absence of immune reconstitution in relapsed patients was noted even in patients receiving secondary prophylaxis (15). Thus, other factors, in addition to the virus and the parasite itself, could contribute to the poor clinical prognosis of patients with VL/HIV co-infection. Finally, additional studies are necessary to confirm the effectiveness of secondary prophylaxis in severely immunocompromised patients.

## Immune activation and inflammatory status in patients with VL/HIV co-infection

In patients with VL/HIV co-infection, chronic immune activation and severe immunosuppression may be potentiated (13, 58, 61, 86), which is an important cofactor in immunological impairment (15, 16, 87). In addition to favoring VL relapse, the degree of immune activation and inflammatory status may be associated with the occurrence of atypical manifestations of VL, such as cutaneous dissemination or PKDL (67, 88), or even progression to severe VL in co-infected patients (58) as well as in patients without HIV infection (37).

We had shown for the first time that patients with VL/HIV co-infection in VL clinical remission present with high percentages of CD8^+^ T cells expressing CD38 (61). Furthermore, these activated T cells were associated with low counts of CD4^+^ T lymphocytes regardless of the use of cART, undetectable viral loads, or clinical remission of VL due to anti-*Leishmania* treatment (61). This cellular activation status was later confirmed in patients with VL/HIV co-infection in the active phase of VL, when patients already had a low or undetectable parasite load combined with effective viral control (13). Subsequently, other studies confirmed high percentages of activated CD8^+^ T lymphocytes (CD38^+^HLA-DR^+^) in Brazilian or Spanish co-infected patients with asymptomatic VL (86) or previous history of VL relapses (87), respectively.

The presence of high serum cytokine levels has also been investigated as a predictor of the clinical progression of VL (37, 58, 89–91). Indeed, similar to T-cell activation, the plasma levels of pro-inflammatory cytokines (IFN-γ, IL-6, IL-8, TNF, and MIP-1β) were higher in patients with VL/HIV co-infection than in those with HIV or VL mono-infection and healthy individuals (13). More recently, IFN-γ and TNF levels have been correlated with severity and death as well as clinical findings such as vomiting and dyspnea in patients with VL/HIV co-infection (58).

In this scenario, long-term follow-up of one patient with VL/HIV co-infection who presented with VL relapse with cutaneous manifestations three months post-therapy showed that this episode was associated with an increase in T-cell activation (67). These data provide the first evidence of a relationship between immune activation and VL relapse. Subsequently, in the cohort evaluated up to 12 months post-treatment, patients with non-relapsing and relapsing VL/HIV showed similar levels of activation of CD4^+^ and CD8^+^ T cells (CD38^+^HLA-DR^+^ expression) in the active phase of VL, but only those with non-relapsing VL/HIV showed a long-term reduction in these percentages post-treatment (15). Patients with relapsing VL/HIV also showed persistently higher levels of pro-inflammatory cytokines (IL-8, TNF, IFN-γ, IL-6, IL-1β, and others) and IL-10 than those with non-relapsing VL/HIV, who tended to show gradual reductions in the levels of these cytokines after treatment (16).

Since patients with relapsing VL/HIV show a high inflammatory status even under effective anti-*Leishmania* treatment and cART, *L. infantum* infection can be plausibly considered to not be the sole factor responsible for enhancing immune activation in HIV-infected individuals.

## Microbial translocation as an additional factor influencing immune activation in VL/HIV co-infection

Microbial translocation from the intestinal lumen to the bloodstream, a phenomenon known to occur in HIV infection (92–94) and already evidenced in VL (31, 90), could constitute another important cofactor for maintaining a high degree of activation in VL/HIV patients (**Figure 1**). In HIV or simian immunodeficiency virus (SIV) infections, damage to the intestinal barrier, characterized by the death of enterocytes resulting in increased permeability, can be mediated by viral replication itself. Indirect mechanisms such as massive destruction of memory CD4^+^CCR5^+^ T cells present in the intestinal mucosa and/or loss of IL-17-producing cells may also contribute to this damage, since they are crucial in the response to bacterial antigens through the neutrophil infiltration, and maintenance of intestinal homeostasis (92, 93, 95–99). As a result, HIV-infected patients present symptoms characteristic of enteropathy, such as diarrhea, malabsorption, inflammatory infiltrates, villus atrophy, and crypt hyperplasia in the mucous tissue (100). Thus, this evidence favors the translocation of bacterial products into systemic circulation (100), constituting one of the main immunopathogenic mechanisms associated with HIV infection (93, 101, 102).

The following molecules have become hallmarks of chronic immune stimulation due to microbial translocation: intestinal fatty acid binding protein (I-FABP) (103), lipopolysaccharide (LPS) from gram-negative bacteria, and soluble receptor CD14 (sCD14) (92, 104, 105). Bacterial components stimulate the cells involved in innate immunity through Toll-like receptor (TLR) ligands (106). LPS, for example, binds to its receptor CD14 and the TLR4-MD2 complex, culminating in the activation of the transcriptional factor NF-κB and the production of inflammatory cytokines such as IL-6, IL-1β, TNF, and IFN type-I. As mentioned previously, these cytokines contribute to the persistent local and systemic immune activation observed during the chronic phase of HIV infection, resulting in a vicious circle. Moreover, elevated LPS levels have been linked to high indices of immune activation in CD8^+^ T cells in HIV-infected patients (92, 104, 107).

Amastigote forms observed in the mucosa-associated lymphoid tissue (MALT) of patients with VL (108, 109) can lead to intestinal damage. Translocation of microbial products, which is implied by increased plasma levels of LPS, its sCD14 receptor, and I-FABP, has been observed in patients with active VL (31). Such elevated LPS levels are correlated with T-cell activation and high levels of pro-inflammatory cytokines such as macrophage migration inhibitory factor (MIF) and IL-8 (31). In patients with VL/HIV, LPS levels are positively correlated with sCD14 levels, specifically in patients with low CD4^+^ counts (<200 cells/mL) (58), as well as with the percentages of activated CD8^+^ T lymphocytes and IL-6 and IL-8 levels (13). The levels of these molecules have also been shown to be significantly increased in patients with previous VL in comparison with those showing an immunodiscordant response to cART (IDR; CD4 count < 200 cells/μL) without VL (87). In this context, a recent study showed that sCD14 is the only independent predictor of disease severity and death in VL/HIV co-infection (58). Furthermore, the levels of other soluble factors associated with microbial translocation and intestinal damage, such as MIF and I-FABP, have also been shown to be elevated in patients with VL/HIV, both in the active phase and in remission (13).

In terms of relapse, high levels of sCD14 were found in relapsing VL/HIV patients even at 12 months post-treatment, while the levels in non-relapsing patients decreased immediately after treatment (15). Interestingly, sCD14 levels negatively correlated with the absolute counts of CD4^+^ T cells, corroborating the profile of T-cell activation (CD38^+^HLA-DR^+^) in these groups (15).

This phenomenon was recently demonstrated in an elegant VL experimental model study (110), in which intestinal dysbiosis was induced in mice and hamsters by long-term treatment with broad-spectrum antibiotics (110). Weight loss, splenomegaly, and hepatomegaly were significantly less severe in the antibiotic-treated infected hamsters than in untreated animals (110), suggesting that pathobionts contribute to disease progression. Using a different approach, our group observed that infected golden hamsters show intestinal changes and evidence of bacterial translocation by increased plasma levels of LPS (111). Moreover, we verified that in comparison with untreated infected animals, infected animals treated with antimonial and amikacin showed significant reductions in the levels of LPS and activated CD4^+^CD25^+^ T cells at 60 days post-infection (dpi), as well as an increase in the percentage of CD4^+^ T cells at 120 dpi (111). These results provide empirical evidence for the benefits of combining VL treatment with antibiotics in affected patients.

These findings also reinforce the idea that microbial translocation can contribute to the maintenance of an intense degree of cellular activation and pro-inflammatory response, and can, therefore, directly influence the impairment of the immune response necessary for parasite control (112) (**Figure 1**). In other words, microbial translocation may be an additional mechanism associated with clinical outcomes such as the severity and relapse of VL in patients co-infected with HIV.

Several mechanisms underlying the activated immune status may directly affect the effector function of T lymphocytes, either quantitatively or qualitatively. Thus, similar to the enhanced degree of activation in a VL/HIV association scenario, the immunological consequences of this process may also be intensified. This hypothesis is based on the fact that each infection commonly involves a process of chronic failure of the immune system, which has been well-characterized in HIV infection and more recently in VL.

## Immunological consequences of chronic immune activation and its influence on the immunopathogenesis of VL/HIV co-infection

The deterioration of immune competence that occurs with aging is a natural process that results from successive moments of cellular activation. This process partially explains the increased morbidity and mortality among elderly individuals without pathological immunodeficiencies (113). Similarly, the intense immune activation in HIV infection has been shown to result in faster progression to immunological aging (114). Consequently, these individuals exhibit severe and early immunological impairments that usually manifest only in the elderly population (114). This process, called immunosenescence, can be clonal, with the functional loss of virus-specific clones, and/or global, with the exhaustion of central immune compartments such as the thymus and bone marrow (115).

Pro-inflammatory cytokines, such as TNF, IL-1β and IL-6, are secreted in response to various infections and tissue damage, and their secretion constitutes a complex initial cascade associated with pathogen destruction and tissue repair, which acts as the natural response to these stressful situations. However, in patients with VL or HIV infection, excessive production and/or accumulation of these mediators results in severe immunological damage. This process, which is known as inflammaging, is characterized by the hyperregulation of anti-stress responses and the production of pro-inflammatory cytokines (113). The combination of inflammaging with immunosenescence has been described to aggravate the degree of immunodeficiency in HIV infection (116, 117).

Immunosenescence is characterized by the presence of numerous clones of terminally differentiated CD4^+^ and CD8^+^ T cells (118). CD57^+^ T cells (118–120) and telomere shortening (114, 118) are widely used to define replicative senescence. In addition to the loss of replicative capacity, CD57^+^ T cells exhibit increased susceptibility to activation-induced cell death (119, 121). Depending on the specificity lost as a consequence of HIV infection (114–116, 122–125), patients may show loss of viral load control, faster progression to AIDS, and impairment of the immune response to other pathogens, such as *Leishmania* spp., which would be critical in the context of VL/HIV co-infection.

VL/HIV patients with and without active disease show higher percentages of senescent T cells, mainly of the CD8^+^ T-cell subpopulation (15, 86, 87), in comparison with patients showing HIV mono-infection, reinforcing the augmented chronically activated immune status in this association (61, 67). Despite these findings, few studies have investigated the degree of immunosenescence in VL and its consequences for the clinical outcomes of the disease.

Cellular exhaustion, which has also been explored in both infections, is phenotypically characterized by the expression of inhibitory molecules such as programmed cell death protein 1 (PD-1), cytotoxic T-lymphocyte–associated antigen 4 (CTLA-4), lymphocyte-activation gene 3 (LAG-3), and T-cell immunoglobulin and mucin domain-containing protein 3 (TIM-3). Both PD-1 and CTLA-4 negatively regulate T-cell activation and are characteristic markers of T-cell anergy/exhaustion during chronic infections (126). Similar to replicative senescence, but in a reversible manner, immune exhaustion results in decreased cytokine-production capacity (IL-2, TNF, and IFN-γ), as well as reduced proliferative capacity (126, 127).

Similar to HIV infection (123, 128–130), the high degree of cellular activation in VL may worsen the clinical condition of these patients by contributing to their exhaustion status. This phenomenon has been identified in human VL (126), canine VL (131, 132), and experimental VL (133) by increasing the phenotypic expression of PD-1 and CTLA-4, particularly in CD8^+^ T lymphocytes. In addition, specific functional impairment due to decreased proliferative capacity and IFN-Ɣ production in response to *L. infantum* antigens has been described (134). However, PD-1/PD-L1 pathway (PD-1 ligand) blockade can reverse this scenario, increasing the proliferative capacity against the specific antigen, restoring the Th1 response, and allowing the production of IFN-γ and increasing cytotoxic activity (133, 135). Thus, blocking these inhibitory molecules may be a promising therapeutic strategy to partially restore the immunological status of chronically infected individuals.

Although few studies have evaluated the role of these inhibitors in VL/HIV co-infection, Ethiopian VL/HIV patients, even under cART, showed high levels of T-cell immunoreceptor with Ig and ITIM domains (TIGIT) and PD1 on CD8-positive and CD8-negative T cells, along with reduced T-cell functionality as a result of the lower frequency of IFN-γ^+^ on TIGIT^+^ T cells (136). Similarly, the impaired specific production of IFN-γ seen in patients with VL/HIV co-infection may be related to the low CD4^+^ T-cell counts and the persistent activation/inflammation and exhaustion of T cells (33). In addition to elucidating the pathogenic mechanisms, future studies should aim to better explore the role of these molecules as possible predictors of VL severity and relapse.

The consequences of immune activation may not be limited to the loss of specific T-cell clones and phenotypic characteristics. Considering their oligoclonal and senescent state, these “terminally differentiated” cells are unlikely to be replaced by a pool of new naive T cells capable of responding to infection (137, 138). In this scenario, the exhaustion of primary immune resources is considered an important cofactor for the maintenance of this immunosenescent state in chronic infections such as HIV. Indeed, HIV-positive patients show impaired bone marrow function with loss of lymphocytic progenitors and thymic atrophy, leading to changes in immunological homeostasis and an inability to reconstitute the T and B cell compartments (139–142). This entire process can culminate in an imbalance between the specific immune response and residual viremia, resulting in the appearance and/or reappearance of other pathologies that characterize AIDS.

The thymus plays a central role in the generation of new T cells and immune reconstitution (139, 143, 144). Infection and death of thymocytes and thymic stromal cells by HIV, infection of hematopoietic stem cells, accelerated thymic atrophy, effects of pro-inflammatory cytokines (TNF), and intense degree of activation are among the factors involved in the impaired thymic function (143, 145–148). Recent thymic emigrants (RTE), newly generated T cells exported from the thymus to the periphery, can be identified by quantification of signal joint T-cell receptor (TCR) rearrangement excision circles (sjTREC) by real-time polymerase chain reaction (139). This assay involves quantification of episomal DNA that is generated during the rearrangement process of TCR genes (139) and is present mainly in cells that express TCR-αβ (Tαβ cells) (139). In addition, the CD31 molecule (PECAM-1) has also been commonly used for evaluating RTE by flow cytometry. This is because the sjTREC content is higher in naive CD4^+^ T cells expressing CD31, and the decline of these CD31^+^CD4^+^ T cells and TRECs levels occurs with aging (149).

Both HIV-infected humans and SIV-infected monkeys show a decrease in TREC content in naive peripheral blood T cells (139, 150–155). This decrease in thymic output is correlated with low immunological reconstitution and, consequently, low CD4^+^ T-cell counts (156, 157), possible loss of viremia control (158), and poor clinical prognosis in HIV infections.

However, the role of the thymus in the immunopathogenesis of VL has not yet been fully explored. Protein malnutrition in *L. infantum*-infected mice significantly alters the thymic microenvironment (22, 159). Atrophy, hypocellularity, and changes in the migration patterns of T-lymphocyte subpopulations were observed, in addition to reductions in the cortical area and intrathymic proliferation (22, 159). More recently, amastigote forms were found in the thymus of *L. infantum-*infected dogs (23).

Patients with VL may present with multifactorial injury of the T-lymphocyte lineage (1): impairment of progenitors in the bone marrow by parasitism (2), natural thymic involution that occurs with aging, and (3) thymic dysfunction induced by malnutrition, as well as the consequences of the infection itself, such as cellular activation (**Figure 1**).

Consequently, in the context of VL/HIV co-infection, simultaneous thymic impairment may contribute to the severity of this association. We demonstrated, for the first time, that patients with VL/HIV co-infection had lower levels of sjTRECs than those with HIV mono-infection, even under undetectable viral loads (16). Interestingly, patients with relapsing VL/HIV co-infection showed low levels of sjTRECs throughout the prospective follow-up period, whereas those with non-relapsing VL/HIV co-infection showed a significant increase at 10 months post-treatment (16), suggesting that thymic impairment may be related to the clinical outcome of VL in patients with HIV co-infection.

Disturbances in the T-lymphocyte repertoire are another qualitative consequence of immunosenescence. After thymic rearrangement, maturation, and selection, T cells migrate to the periphery. Thus, disorders of thymic function profoundly affect the diversity of the T-lymphocyte repertoire and, consequently, the capacity to respond to a variety of antigens (160). Assessments of TCR diversity are currently based on flow-cytometry and next-generation sequencing studies of the families that constitute the variable region of the β chain (Vβ). Disturbances in the Vβ repertoire have been related to the immunopathogenesis of several diseases, such as cancer (161, 162), Chagas disease (163, 164), HIV/AIDS (165–167), and leishmaniasis (16, 168, 169). Studies on American Tegumentary Leishmaniasis (ATL) showed expansion of the Vβ12 and Vβ22 families and contraction of Vβ2 in *L. braziliensis-*infected patients (169). Moreover, a decrease in CD8^+^Vβ14^+^ T cells in the lymph nodes has been observed (168), in contrast to the augmentation of this family in the lesions of *L. guyanensis-*infected patients (170), indicating the migration of these cells among immune compartments (170).

In relation to VL/HIV co-infection, unprecedentedly, our group demonstrated the occurrence of significant disorders of the Vβ repertoire (16). In this study, in comparison with healthy individuals, patients with relapsing VL/HIV co-infection showed a more heterogeneous Vβ repertoire mobilization profile throughout the follow-up period, especially in the CD8^+^ T cells, in terms of expansion and retraction of Vβ families. In contrast, patients with non-relapsing VL/HIV co-infection presented a profile with significant changes, although specific to certain families. The Vβ3 and Vβ18 families were less expressed in CD8^+^ T cells from patients with relapsing VL/HIV co-infection and expanded among patients with non-relapsing VL/HIV co-infection, especially in the active phase of the disease. Despite these findings, no characteristic profile of the dynamics of the TCRVβ repertoire in terms of clonality was observed in this study, making it impossible to associate it with clinical outcomes in terms of relapses (16).

Finally, although barely investigated, these results point to profound disturbances in the immune compartments due to associations between two parasites whose effects are progressively potentialized as a result of sharing very similar immunopathological mechanisms. Thus, prompt diagnosis and treatment of both diseases can certainly prevent more severe consequences of VL/HIV co-infection.

## Relapses of VL in patients with or without HIV co-infection: potential biomarkers

According to the Brazilian Ministry of Health, VL relapses are characterized by resurgence of symptoms within 12 months of clinical cure (11). As described by Cota et al., relapses present clinically with the reappearance of fever, worsening cytopenia, or an increase in splenomegaly after successful drug treatment (82, 171). Relapse is a very common outcome among immunosuppressed patients, such as those co-infected with HIV and transplant recipients (15, 16, 172, 173), although its incidence is also increasing among individuals without other comorbidities (32).

Horrillo et al. described treatment failures and reported that the prevalence of VL relapse was 12%, with the relapses being associated with a lack of adequate prophylaxis in patients co-infected with HIV and liposomal amphotericin B doses lower than 21 mg/kg in patients without HIV (174). Abongomera et al. described data from an Ethiopian cohort of patients with VL/HIV co-infection, in which 35% of the individuals showed relapse. cART is associated with a lower risk of relapse, whereas high parasite loads are associated with disease recurrence (175). Similarly, other studies have demonstrated that the blood parasite load (176), male sex, extremes of age (<5 and >45 years), and a slight decrease in splenomegaly (177) are risk factors for relapse in patients with VL without HIV. A recent study demonstrated that, in addition to HIV infection, factors such as thrombocytopenia, lower limb edema, and secondary pneumonia were independently associated with relapse (173).

Several studies have already shown that low CD4^+^ T lymphocyte counts during the active phase of VL or even the absence of an increase in this subpopulation after treatment can be considered a predictive factor for VL relapse in HIV-infected patients (178–184). In this way, previous studies by our group have demonstrated that the number of VL episodes is inversely correlated with the CD4^+^ T-cell count and sjTREC level in patients with VL/HIV co-infection (15, 16) and could be a useful immunological biomarker for disease relapse. Interestingly, patients with relapsing VL without a history of HIV also maintained low CD4 T-cell counts post-treatment (32). These studies reinforce the relationship between the degree of immunological reconstitution and different clinical outcomes of the disease.

Bhattacharyya et al. showed that high levels of anti-SLA (soluble *Leishmania* antigen) immunoglobulin (Ig)G1 at 6 months post-treatment were associated with treatment failure and relapse (185), demonstrating the importance of IgG1 in determining the clinical status of patients with VL, without HIV. These findings were confirmed by subsequent studies that demonstrated the possibility of using IgG1 anti-rK39 antibodies as biomarkers for VL relapse (186, 187). Similarly, Mondal et al. demonstrated the potential of quantifying serum IgG anti-rK39 antibodies to determine VL prognosis (188). Corroborating these data, our group demonstrated that patients with several episodes of VL showed high levels of anti-SLA immunoglobulins, especially IgG3, even 12 months post-treatment and independent of the HIV serological status (15, 32). These studies point to IgG3 as a possible biomarker of relapse, and indicate that a reduction in its serum levels may be related to the clinical remission of VL.

**Figure 2:**
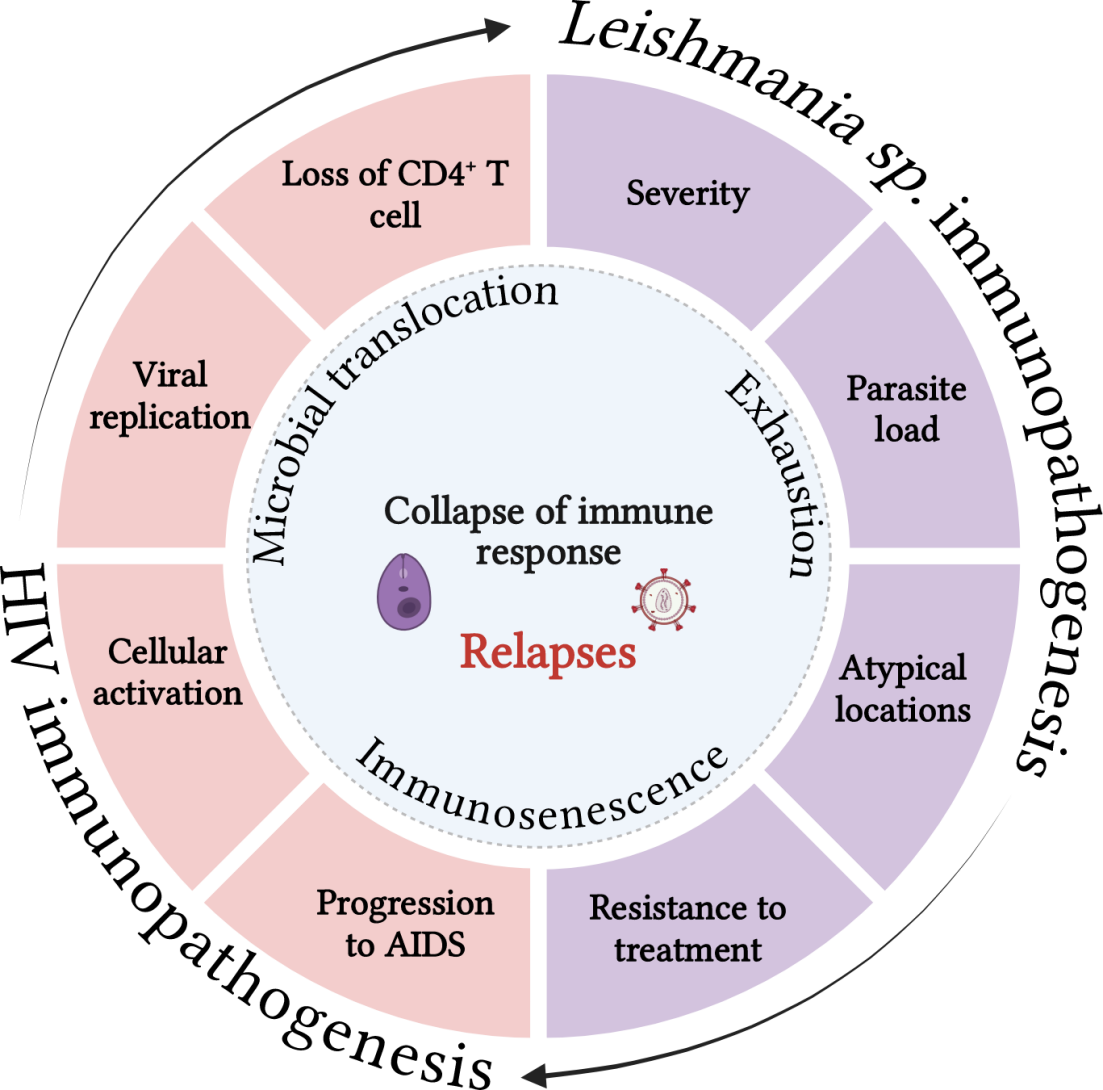
Both infections share immunopathogenic characteristics that can reciprocally impair the immune response to pathogens. Visceral leishmaniasis can contribute to the decrease in CD4 T lymphocytes, worsening immunosuppression, increasing viral replication and the cellular activation degree, which in turn may favor progression to AIDS. On the other hand, HIV infection and its consequences for the immune response can increase the parasite load, the resistance to the treatment, dissemination of VL for atypical sites and favor the severity and relapses of the disease. Microbial translocation, immunosenescence and the exhaustion degree may act as key cofactors of the process that culminates in the collapse of the immune response in co-infected patients. Source: The author. BioRender.com.

In conclusion, despite the considerable prevalence of VL relapse in patients co-infected with HIV and, more recently, in immunocompetent patients, official protocols for recognizing patients susceptible to relapses are lacking, and their therapeutic management is not well-defined. This scenario, combined with the fact that available treatments are scarce, have significant toxicity, and pose a high cost to the health system, highlight the need to investigate the reactivation-related immunological mechanisms that can help predict the clinical prognosis of these patients (**Figure 2**). Finally, microbial translocation (sCD14), exhaustion (PD-1/TIGIT)- and senescence (TREC)-associated markers along with immune activation profile (IgG1/IgG3 and CD38/HLA-DR) deserve to be better investigated as underlying determinants of relapse or chronicity of VL in patients with HIV co-infection, in addition to being evaluated using algorithms to validate them as prognostic biomarkers (**Figure 1**, **Table 1**). Finally, the cross-sectional studies on VL/HIV conducted to date have provided evidence of the global immunological impairment experienced by these patients. However, prospective multicenter studies are crucial to investigate which immunological parameters can be good predictors of prognosis in terms of VL relapse and/or severity in patients with VL/HIV co-infection.

**Table 1:** Potential biomarkers related to severity, death or relapses of VL in HIV co-infected patients.

Immunopathogenic mechanism	Biomarkers	Relapses	Severity and Death
*Immune activation*	Cytokine storm	16	58
	CD38 and HLA-DR on T cells	15, 67	–
	Parasite load	175	–
	Anti-*Leishmania* IgG3	15	–
	Soluble CD14	15	58
*Deficient immune reconstitution*	CD4^+^ T cells absolute counts	12, 15, 62, 178-184	184
	sjTREC	16	–
	Vβ disturbances	16	–
*Immunosuppression*	Antigen-specific IFN-γ	62	–
	Anergy/Exhaustion	62, 136	–

## Glossary

VL: visceral leishmaniasis
HIV: human immunodeficiency virus
VL/HIV: visceral leishmaniasis and human immunodeficiency virus co-infection
AIDS: acquired immunodeficiency syndrome
cART: combined antiretroviral therapy
CD: cluster of differentiation
IL: interleukin
IFN: interferon
TNF: tumor necrosis factor
RANTES: normal T-cell expressed and secreted
MIP: macrophage inflammatory protein
CXCL: chemokine ligand
NF: nuclear factor
MDM: monocyte-derived macrophages
PI: protease inhibitor
IRIS: immune reconstitution inflammatory syndrome
PKDL: post-kala-azar dermal leishmaniasis
HLA: Human Leucocyte Antigen
SIV: simian immunodeficiency virus
CCR: chemokine receptor
I-FABP: intestinal fatty-acid binding protein
LPS: lipopolysaccharide
sCD14: soluble receptor CD14
TLR: Toll-like receptor
MALT: mucosa-associated lymphoid tissue
MIF: macrophage migration inhibitory factor
PD-1: programmed cell death protein 1
CTLA-4: cytotoxic T-lymphocyte–associated antigen 4
LAG-3: lymphocyte-activation gene 3
TIM-3: T-cell immunoglobulin and mucin domain-containing protein 3
PD-L1: programmed cell death protein 1 ligant
TIGIT: T-cell immunoreceptor with Ig and ITIM domains
RTE: Recent thymic emigrants
TCR: T-cell receptor
sjTREC: signal joint TCR rearrangement excision circles
PECAM-1: platelet endothelial cell adhesion molecule 1
Vβ: variable region of the β
ATL: American Tegumentary Leishmaniasis
Ig: immunoglobulin

## References

[1] Leite de Sousa-Gomes M Romero GAS Werneck GL. Visceral leishmaniasis and HIV/AIDS in Brazil: Are we aware enough? PloS Negl Trop Dis. (2017) 11:1–13. doi: 10.1371/journal.pntd.0005772 PMC5612457 28945816

[2] Graepp-Fontoura I Barbosa DS Fontoura VM Guerra RNM Melo AS Fernandes MNF. Visceral leishmaniasis and HIV coinfection in Brazil: epidemiological profile and spatial patterns. Trans R Soc Trop Med Hyg. (2023) 117:260–70. doi: 10.1093/trstmh/trac093 36219448

[3] World Health Organization. Leishmaniasis: Fact sheet 2023 (2023). Available online at: https://www.who.int/news-room/fact-sheets/detail/leishmaniasis (Accessed Aug 30, 2023).

[4] De La Loma A Alvar J Martinez Galiano E Blázquez J Alcalá Muñoz A Nájera R. Leishmaniasis or AIDS? Trans R Soc Trop Med Hyg. (1985) 79:421–2. doi: 10.1016/0035-9203(85)90400-6 4035745

[5] Cruz I Nieto J Moreno J Cañavate C Desjeux P Alvar J. *Leishmania*/HIV co-infections in the second decade. Indian J Med Res. (2006) 123:357–88. 16778317

[6] Desjeux P Alvar J. *Leishmania*/HIV co-infections: epidemiology in europe. Ann Trop Med Parasitol. (2003) 97:3–15. doi: 10.1179/000349803225002499 14678629

[7] World Health Organization. Number of cases of visceral leishmaniasis reported 2022 (2022). Available online at: https://www.who.int/data/gho/data/indicators/indicator-details/GHO/number-of-cases-of-visceral-leishmaniasis-reported (Accessed Aug 30, 2023).

[8] Pineda JA Macías J Morillas F Fernandez-Ochoa J Cara J de la Rosa R. Evidence of increased risk for *Leishmania infantum* infection among HIV-seronegative intravenous drug users from southern Spain. Eur J Clin Microbiol Infect Dis. (2001) 20:354–7. doi: 10.1007/PL00011276 11453599

[9] Ministério da Saúde (BR). Leishmaniose visceral (2023). Available online at: https://www.gov.br/saude/pt-br/assuntos/saude-de-a-a-z/l/leishmaniose-visceral/arquivos/lv-graficos-e-mapas.pdf (Accessed Aug 30, 2023).

[10] Lindoso JA Moreira CHV Cunha MA Queiroz IT. Visceral leishmaniasis and HIV coinfection: current perspectives. HIV AIDS (Auckl). (2018) 10:193–201. doi: 10.2147/HIV.S143929 30410407 PMC6197215

[11] Ministério da Saúde (BR). Manual de recomendações para diagnóstico, tratamento e acompanhamento de pacientes com a coinfecção *Leishmania*-HIV (2015). Available online at: https://www.gov.br/saude/pt-br/centrais-de-conteudo/publicacoes/svsa/leishmaniose/manual-de-recomendacoes-para-diagnostico-tratamento-e-acompanhamento-de-pacientes-com-a-coinfeccao-leishmania-hiv.pdf/view (Accessed Aug 30, 2023).

[12] Cota GF de Sousa MR Rabello A. Predictors of visceral leishmaniasis relapse in HIV-infected patients: A systematic review. PloS Negl Trop Dis. (2011) 5:1–8. doi: 10.1371/journal.pntd.0001153 PMC3110161 21666786

[13] Santos-Oliveira JR Regis EG Giacoia-Gripp CBW Valverde JG Alexandrino-De-Oliveira P Lindoso JÂL. Microbial translocation induces an intense proinflammatory response in patients with visceral leishmaniasis and HIV type 1 coinfection. J Infect Dis. (2013) 208:57–66. doi: 10.1093/infdis/jit135 23539743

[14] Okwor I Uzonna JE. The immunology of *Leishmania*/HIV co-infection. Immunol Res. (2013) 56:163–71. doi: 10.1007/s12026-013-8389-8 23504228

[15] Silva-Freitas ML Cota GF MaChado-De-Assis TS Giacoia-Gripp C Rabello A Da-Cruz AM. Immune activation and bacterial translocation: A link between impaired immune recovery and frequent visceral leishmaniasis relapses in HIV-infected patients. PloS One. (2016) 11:1–18. doi: 10.1371/journal.pone.0167512 PMC5132299 27907136

[16] Silva-Freitas ML Corrêa-Castro G Cota GF Giacoia-Gripp C Rabello A Teixeira Dutra J. Impaired thymic output can be related to the low immune reconstitution and T cell repertoire disturbances in relapsing visceral leishmaniasis associated HIV/AIDS patients. Front Immunol. (2020) 11:1–14. doi: 10.3389/fimmu.2020.00953 32508833 PMC7251171

[17] Lindoso JA Cota GF Da-Cruz AM Goto H Maia-Elkhoury ANS Romero GAS. Visceral leishmaniasis and HIV coinfection in Latin America. PloS Negl Trop Dis. (2014) 8:1–9. doi: 10.1371/journal.pntd.0003136 PMC4169383 25233461

[18] Sarkari B Naraki T Ghatee MA Khabisi AS Davami MH. Visceral leishmaniasis in southwestern Iran: A retrospective clinico-hematological analysis of 380 consecutive hospitalized cases (1999-2014). PloS One. (2016) 11:1–8. doi: 10.1371/journal.pone.0150406 PMC4778872 26942443

[19] Shiferaw E Murad F Tigabie M Abebaw M Alemu T Abate S. Hematological profiles of visceral leishmaniasis patients before and after treatment of anti-leishmanial drugs at University of Gondar Hospital; *Leishmania* Research and Treatment Center Northwest, Ethiopia. BMC Infect Dis. (2021) 21:1–7. doi: 10.1186/s12879-021-06691-7 34565339 PMC8474942

[20] Debash H Bisetegn H Nigatie M Abeje G Feleke DG. Epidemiological, clinical and hematological profiles of visceral leishmaniasis among patients visiting Tefera Hailu Memorial Hospital, Northeast Ethiopia: a 4 year retrospective study. Sci Rep. (2023) 13:1–10. doi: 10.1038/s41598-023-28139-5 36650391 PMC9845332

[21] Veras PST de Santana MBR Brodskyn CI Fraga DBM Solcà MS De Menezes JPB. Elucidating the role played by bone marrow in visceral leishmaniasis. Front Cell Infect Microbiol. (2023) 13:1–13. doi: 10.3389/fcimb.2023.1261074 PMC10582953 37860064

[22] Losada-Barragán M Umaña-Pérez A Durães J Cuervo-Escobar S Rodríguez-Vega A Ribeiro-Gomes FL. Thymic microenvironment is modified by malnutrition and *Leishmania infantum* infection. Front Cell Infect Microbiol. (2019) 9:1–19. doi: 10.3389/fcimb.2019.00252 31355153 PMC6639785

[23] da Silva AVA de Souza TL Figueiredo FB Mendes AAV Ferreira LC Filgueira CPB. Detection of amastigotes and histopathological alterations in the thymus of *Leishmania infantum*-infected dogs. Immun Inflammation Dis. (2020) 8:127–39. doi: 10.1002/iid3.v8.2 PMC7212199 32207879

[24] Savino W Durães J Maldonado-Galdeano C Perdigon G Mendes-da-Cruz DA Cuervo P. Thymus, undernutrition, and infection: approaching cellular and molecular interactions. Front Nutr. (2022) 9:1–20. doi: 10.3389/fnut.2022.948488 PMC9549110 36225882

[25] Fontes JLM Khouri R Reinaldo LGC Hassegawa EMA Meneses Filho AJ de Melo CVB. An integrated analysis of the structural changes and gene expression of spleen in human visceral leishmaniasis with and without HIV coinfection. PloS Negl Trop Dis. (2024) 18:1–16. doi: 10.1371/journal.pntd.0011877 PMC11265696 38843306

[26] Caldas A Favali C Aquino D Vinhas V van Weyenbergh J Brodskyn C. Balance of IL-10 and interferon-gamma plasma levels in human visceral leishmaniasis: implications in the pathogenesis. BMC Infect Dis. (2005) 5:113. doi: 10.1186/1471-2334-5-113 16364177 PMC1343567

[27] Goto H Prianti MDG. Immunoactivation and immunopathogeny during active visceral leishmaniasis. Rev Inst Med Trop Sao Paulo. (2009) 51:241–6. doi: 10.1590/S0036-46652009000500002 19893975

[28] Silva LA Romero HD Nascentes GAN Costa RT Rodrigues V Prata A. Antileishmania immunological tests for asymptomatic subjects living in a visceral leishmaniasis-endemic area in Brazil. Am J Trop Med Hyg. (2011) 84:261–6. doi: 10.4269/ajtmh.2011.10-0092 PMC3029179 21292896

[29] Carvalho EM Badaro R Reed SG Jones TC Johnson WD. Absence of gamma interferon and interleukin 2 production during active visceral leishmaniasis. J Clin Invest. (1985) 76:2066–9. doi: 10.1172/JCI112209 PMC424308 3935667

[30] Soong L Henard CA Melby PC. Immunopathogenesis of non-healing american cutaneous leishmaniasis and progressive visceral leishmaniasis. Semin Immunopathol. (2012) 34:735–51. doi: 10.1007/s00281-012-0350-8 PMC4111229 23053396

[31] Santos-Oliveira JR Regis EG Leal CRB Cunha RV Bozza PT Da-Cruz AM. Evidence that lipopolisaccharide may contribute to the cytokine storm and cellular activation in patients with visceral leishmaniasis. PloS Negl Trop Dis. (2011) 5:1–10. doi: 10.1371/journal.pntd.0001198 PMC3134430 21765960

[32] Kuschnir RC Pereira LS Dutra MRT de Paula L Silva-Freitas ML Corrêa-Castro G. High levels of anti-*Leishmania* igG3 and low CD4+ T cells count were associated with relapses in visceral leishmaniasis. BMC Infect Dis. (2021) 21:1–14. doi: 10.1186/s12879-021-06051-5 33874901 PMC8056614

[33] Rodrigues-Neto JF Monteiro GR Keesen TSL Lacerda HG Carvalho EM Jeronimo SMB. CD45RO+ T cells and T cell activation in the long-lasting immunity after *Leishmania infantum* infection. Am J Trop Med Hyg. (2018) 98:875–82. doi: 10.4269/ajtmh.16-0747 PMC5930877 29280433

[34] Peruhype-Magalhães V Martins-Filho OA Prata A Silva LA Rabello A Teixeira-Carvalho A. Mixed inflammatory/regulatory cytokine profile marked by simultaneous raise of interferon-gamma and interleukin-10 and low frequency of tumour necrosis factor-alpha(+) monocytes are hallmarks of active human visceral leishmaniasis due to *Leishmania chagasi* infection. Clin Exp Immunol. (2006) 146:124–32. doi: 10.1111/j.1365-2249.2006.03171.x PMC1809731 16968407

[35] Araújo-Santos T Andrade BB Gil-Santana L Luz NF Dos Santos PL De Oliveira FA. Anti-parasite therapy drives changes in human visceral leishmaniasis-associated inflammatory balance. Sci Rep. (2017) 7:1–8. doi: 10.1038/s41598-017-04595-8 28659627 PMC5489532

[36] Costa CHN Werneck GL Costa DL Holanda TA Aguiar GB Carvalho AS. Is severe visceral leishmaniasis a systemic inflammatory response syndrome? A case control study. Rev Soc Bras Med Trop. (2010) 43:386–92. doi: 10.1590/S0037-86822010000400010 20802936

[37] Costa DL Rocha RL Carvalho RMA Lima-Neto AS Harhay MO Costa CHN. Serum cytokines associated with severity and complications of kala-azar. Pathog Glob Health. (2013) 107:78–87. doi: 10.1179/2047773213Y.0000000078 23683334 PMC4001482

[38] Chang CC Crane M Zhou J Mina M Post JJ Cameron BA. HIV and co-infections. Immunol Rev. (2013) 254:114–42. doi: 10.1111/imr.2013.254.issue-1 PMC3697435 23772618

[39] Dhulipalla M Chouhan G. The nexus between *Leishmania* & HIV: Debilitating host immunity and Hastening Comorbid disease burden. Exp Parasitol. (2024) 265:108826. doi: 10.1016/j.exppara.2024.108826 39147120

[40] Sauce D Elbim C Appay V. Monitoring cellular immune markers in HIV infection: from activation to exhaustion. Curr Opin HIV AIDS. (2013) 8:125–31. doi: 10.1097/COH.0b013e32835d08a9 23380653

[41] Paiardini M Müller-Trutwin M. HIV-associated chronic immune activation. Immunol Rev. (2013) 254:78–101. doi: 10.1111/imr.2013.254.issue-1 23772616 PMC3729961

[42] Mzingwane ML Tiemessen CT. Mechanisms of HIV persistence in HIV reservoirs. Rev Med Virol. (2017) 27:1–12. doi: 10.1002/rmv.v27.2 28128885

[43] Taiwo B Barcena L Tressler R. Understanding and controlling chronic immune activation in the HIV-infected patients suppressed on combination antiretroviral therapy. Curr HIV/AIDS Rep. (2013) 10:21–32. doi: 10.1007/s11904-012-0147-3 23225316

[44] Grund B Baker JV Deeks SG Wolfson J Wentworth D Cozzi-Lepri A. Relevance of interleukin-6 and D-dimer for serious non-AIDS morbidity and death among HIV-positive adults on suppressive antiretroviral therapy. PloS One. (2016) 11:1–16. doi: 10.1371/journal.pone.0155100 PMC4865234 27171281

[45] Younas M Psomas C Reynes J Corbeau P. Immune activation in the course of HIV-1 infection: causes, phenotypes and persistence under therapy. HIV Med. (2016) 17:89–105. doi: 10.1111/hiv.2016.17.issue-2 26452565

[46] Richert Q Trajtman A Arroyave L Toews J Becker M Kasper K. Systemic inflammation before and after antiretroviral therapy initiation as a predictor of immune response among HIV-infected individuals in Manitoba. Cytokine. (2017) 91:74–81. doi: 10.1016/j.cyto.2016.12.010 28012378

[47] Mehraj V Ramendra R Isnard S Dupuy FP Lebouché B Costiniuk C. CXCL13 as a biomarker of immune activation during early and chronic HIV infection. Front Immunol. (2019) 10:1–11. doi: 10.3389/fimmu.2019.00289 30846990 PMC6393370

[48] Alvar J Aparicio P Aseffa A Den Boer M Cañavate C Dedet JP. The relationship between leishmaniasis and AIDS: the second 10 years. Clin Microbiol Rev. (2008) 21:334–59. doi: 10.1128/CMR.00061-07 PMC2292576 18400800

[49] Adriaensen W Dorlo TPC Vanham G Kestens L Kaye PM van Griensven J. Immunomodulatory therapy of visceral leishmaniasis in human immunodeficiency virus-coinfected patients. Front Immunol. (2018) 8:1–16. doi: 10.3389/fimmu.2017.01943 PMC5770372 29375567

[50] Zhao C Papadopoulou B Tremblay MJ. *Leishmania infantum* enhances human immunodeficiency virus type-1 replication in primary human macrophages through a complex cytokine network. Clin Immunol. (2004) 113:81–8. doi: 10.1016/j.clim.2004.06.003 15380533

[51] Garg R Barat C Ouellet M Lodge R Tremblay MJ. *Leishmania infantum* amastigotes enhance HIV production in cocultures of human dendritic cells and CD4 T cells by inducing secretion of IL-6 and TNF-alpha. PloS Negl Trop Dis. (2009) 3:1–11. doi: 10.1371/journal.pntd.0000441 PMC2680485 19468304

[52] Andreani G Lodge R Richard D Tremblay MJ. Mechanisms of interaction between protozoan parasites and HIV. Curr Opin HIV AIDS. (2012) 7:276–82. doi: 10.1097/COH.0b013e32835211e9 22418447

[53] Bernier R Barbeau B Tremblay MJ Olivier M. The lipophosphoglycan of *Leishmania donovani* up-regulates HIV transcription in T cells through the nuclear factor-kappaB elements. J Immunol. (1998) 160:2881–8. doi: 10.4049/jimmunol.160.6.2881 9510191

[54] Wolday D Akuffo H Demissie A Britton S. Role of *Leishmania donovani* and its lipophosphoglycan in CD4 T-cell activation-induced human immunodeficiency virus replication. Infect Immun. (1999) 67:5258–64. doi: 10.1128/IAI.67.10.5258-5264.1999 PMC96879 10496904

[55] Garg R Lodge R Descoteaux A Tremblay MJ. *Leishmania infantum* promastigotes reduce entry of HIV-1 into macrophages through a lipophosphoglycan-mediated disruption of lipid rafts. J Infect Dis. (2008) 197:1701–8. doi: 10.1086/588146 18422456

[56] Costa LDLN Cutrim CMS de Almeida Santos G de Lima URS de Sousa TM do Nascimento JR. Higher levels of IL-6 and IL-10 cytokines in visceral leishmaniasis-HIV co-infected patients from Brazilian high endemic area. Cytokine. (2025) 185:156812. doi: 10.1016/j.cyto.2024.156812 39612657

[57] Barbosa Júnior WL Justo AM Aguiar dos Santos AM de Lorena VMB do Carmo RF de Melo FL. Higher levels of TNF and IL-4 cytokines and low miR-182 expression in visceral leishmaniasis-HIV co-infected patients. Parasite Immunol. (2020) 42:1–9. doi: 10.1111/pim.12701 31990371

[58] Ferreira GR Santos-Oliveira JR Silva-Freitas ML Honda M Costa DL Da-Cruz AM. Biomarkers of disease severity in patients with visceral leishmaniasis co-infected with HIV. Cytokine. (2022) 149:1–8. doi: 10.1016/j.cyto.2021.155747 34715475

[59] Vallejo A Abad-Fernández M Moreno S Moreno A Pérez-Elías MJ Dronda F. High levels of CD4+ CTLA-4+ T reg cells and CCR5 density in HIV-infected patients with visceral leishmaniasis. Eur J Clin Microbiol Infect Dis. (2015) 34:267–75. doi: 10.1007/s10096-014-2229-1 25142804

[60] Alexandrino-de-Oliveira P Santos-Oliveira JR Dorval MEC Da-Costa FCB Pereira GROL Cunha RV. HIV/AIDS-associated visceral leishmaniasis in patients from an endemic area in central-west Brazil. Mem Inst Oswaldo Cruz. (2010) 105:692–7. doi: 10.1590/S0074-02762010000500016 20835619

[61] Santos-Oliveira JR Giacoia-Gripp CBW Alexandrino de Oliveira P Amato VS Lindoso JTL Goto H. High levels of T lymphocyte activation in *Leishmania*-HIV co-infected individuals despite low HIV viral load. BMC Infect Dis. (2010) 10:1–16. doi: 10.1186/1471-2334-10-358 21171992 PMC3022832

[62] Takele Y Mulaw T Adem E Shaw CJ Franssen SU Womersley R. Immunological factors, but not clinical features, predict visceral leishmaniasis relapse in patients co-infected with HIV. Cell Rep Med. (2021) 3:100487. doi: 10.1016/j.xcrm.2021.100487 35106507 PMC8784791

[63] Sinha PK Van Griensven J Pandey K Kumar N Verma N Mahajan R. Liposomal amphotericin B for visceral leishmaniasis in human immunodeficiency virus-coinfected patients: 2-year treatment outcomes in Bihar, India. Clin Infect Dis. (2011) 53:91–8. doi: 10.1093/cid/cir521 21890763

[64] Távora LGF Nogueira MB Gomes ST. Visceral leishmaniasis/HIV co-infection in northeast Brazil: evaluation of outcome. Braz J Infect Dis. (2015) 19:651–6. doi: 10.1016/j.bjid.2015.07.004 PMC9425356 26361839

[65] Monge-Maillo B Norman FF Cruz I Alvar J López-Vélez R. Visceral leishmaniasis and HIV coinfection in the Mediterranean region. PloS Negl Trop Dis. (2014) 8:e3021. doi: 10.1371/journal.pntd.0003021 25144380 PMC4140663

[66] Rosenthal E Marty P del Giudice P Pradier C Ceppi C Gastaut J. HIV and *Leishmania* coinfection: A review of 91 cases with focus on atypical locations of leishmania. Clin Infect Dis. (2000) 31:1093–5. doi: 10.1086/cid.2000.31.issue-4 11049794

[67] Santos-Oliveira JR Da-Cruz AM Pires LHS Cupolillo E Kuhls K Giacoia-Gripp CBW. Case report: atypical lesions as a sign of cutaneous dissemination of visceral leishmaniasis in a human immunodeficiency virus-positive patient simultaneously infected by two viscerotropic *Leishmania* species. Am J Trop Med Hyg. (2011) 85:55–9. doi: 10.4269/ajtmh.2011.10-0398 PMC3122343 21734124

[68] Savoia D Scutera S Raimondo S Conti S Magliani W Polonelli L. Activity of an engineered synthetic killer peptide on *Leishmania* major and *Leishmania infantum* promastigotes. Exp Parasitol. (2006) 113:186–92. doi: 10.1016/j.exppara.2006.01.002 16487518

[69] Piccinini M Rinaudo MT Chiapello N Ricotti E Baldovino S Mostert M. The human 26S proteasome is a target of antiretroviral agents. AIDS. (2002) 16:693–700. doi: 10.1097/00002030-200203290-00004 11964525

[70] Demarchi IG Silveira TGV Ferreira ICP Lonardoni MVC. Effect of HIV protease inhibitors on New World *Leishmania* . Parasitol Int. (2012) 61:538–44. doi: 10.1016/j.parint.2012.04.006 22579524

[71] Santos LO Vitório BS Branquinha MH Pedroso e Silva CM Santos ALS D’avila-Levy CM. Nelfinavir is effective in inhibiting the multiplication and aspartic peptidase activity of *Leishmania* species, including strains obtained from HIV-positive patients. J Antimicrob Chemother. (2013) 68:348–53. doi: 10.1093/jac/dks410 PMC3543121 23109184

[72] Tadesse A Hurissa Z. Leishmaniasis (PKDL) as a case of immune reconstitution inflammatory syndrome (IRIS) in HIV-positive patient after initiation of anti-retroviral therapy (ART). Ethiop Med J. (2009) 47:77–9. 19743785

[73] Gois L Badaró R Schooley R Grassi FR. Immune response to *Leishmania* antigens in an AIDS patient with mucocutaneous leishmaniasis as a manifestation of immune reconstitution inflammatory syndrome (IRIS): A case report. BMC Infect Dis. (2015) 15:1–7. doi: 10.1186/s12879-015-0774-6 25645330 PMC4323250

[74] Schleenvoigt BT Ignatius R Baier M Schneider T Weber M Hagel S. Development of visceral leishmaniasis in an HIV(+) patient upon immune reconstitution following the initiation of antiretroviral therapy. Infection. (2016) 44:115–9. doi: 10.1007/s15010-015-0813-7 26123228

[75] Muço E Karruli A Hoxha N Hoxhaj A Kokici M. Visceral leishmaniasis and herpes zoster as a component of syndrome of immune reconstitution inflammatory syndrome in an HIV-positive patient. Case Rep Infect Dis. (2022) 2022:1–4. doi: 10.1155/2022/2784898 PMC8938089 35321085

[76] Sinha S Fernández G Kapila R Lambert WC Schwartz RA. Diffuse cutaneous leishmaniasis associated with the immune reconstitution inflammatory syndrome. Int J Dermatol. (2008) 47:1263–70. doi: 10.1111/j.1365-4632.2008.03804.x 19126013

[77] Davies O Allen F Gruener AM Simons R Graham EM Larbalestier N. Uveitis secondary to leishmaniasis immune reconstitution syndrome in a HIV-positive patient. Int J STD AIDS. (2016) 27:598–600. doi: 10.1177/0956462415588444 26002317

[78] Lai RPJ Meintjes G Wilkinson RJ. HIV tuberculosis-associated immune reconstitution inflammatory syndrome. Semin Immunopathol. (2016) 38:185–98. doi: 10.1007/s00281-015-0532-2 PMC4779131 26423994

[79] Piggott DA Karakousis PC. Timing of antiretroviral therapy for HIV in the setting of TB treatment. Clin Dev Immunol. (2011) 2011:1–10. doi: 10.1155/2011/103917 PMC3017895 21234380

[80] Soeters HM Poole C Patel MR Van Rie A. The effect of tuberculosis treatment at combination antiretroviral therapy initiation on subsequent mortality: A systematic review and meta-analysis. PloS One. (2013) 8:1–9. doi: 10.1371/journal.pone.0078073 PMC3797056 24143260

[81] Van Griensven J Zijlstra EE Hailu A. Visceral leishmaniasis and HIV coinfection: time for concerted action. PloS Negl Trop Dis. (2014) 8:1–2. doi: 10.1371/journal.pntd.0003023 PMC4148210 25166269

[82] Cota G Sousa MR de Mendonça AL Patrocinio A Assunção LS de Faria SR. *Leishmania*-HIV co-infection: clinical presentation and outcomes in an urban area in Brazil. PloS Negl Trop Dis. (2014) 8:2–8. doi: 10.1371/journal.pntd.0002816 PMC3990491 24743472

[83] Diro E Ritmeijer K Boelaert M Alves F Mohammed R Abongomera C. Use of pentamidine as secondary prophylaxis to prevent visceral leishmaniasis relapse in HIV infected patients, the first twelve months of a prospective cohort study. PloS Negl Trop Dis. (2015) 9:1–15. doi: 10.1371/journal.pntd.0004087 PMC4591988 26431253

[84] Camara L Queirós J Ribeiro R Teófilo E. Meglumine antimoniate combination treatment for relapsing kala-azar after treatment and secondary prophylaxis failure with liposomal amphotericin B in two HIV-coinfected patients. BMJ Case Rep. (2019) 12:1–4. doi: 10.1136/bcr-2019-231929 PMC6936531 31848140

[85] Araújo CF Oliveira IBN Silva MVT Pereira LIA Pinto AS Silveira MB. New World *Leishmania* spp. Infection in people living with HIV: concerns about relapses and secondary prophylaxis. Acta Trop. (2021) 224:1–13. doi: 10.1016/j.actatropica.2021.106146 34562423

[86] Mendes-Aguiar CO do Monte Alves M de Albuquerque Lopes Machado A de Góis Monteiro GR Medeiros IM Queiroz JW. T-cell activation, senescence, and exhaustion in asymptomatic HIV/*Leishmania infantum* co-infection. medRxiv [Preprint]. (2023) 03.06.23286828. doi: 10.1101/2023.03.06.23286828

[87] Casado J Abad-Fernández M Moreno S Pérez-Elías M Moreno A Bernardino J. Visceral leishmaniasis as an independent cause of high immune activation, T-cell senescence, and lack of immune recovery in virologically suppressed HIV-coinfected patients. HIV Med. (2015) 16:240–8. doi: 10.1111/hiv.2015.16.issue-4 25604328

[88] Singh AK Das VNR Amit A Dikhit MR Mahantesh V Singh SK. Cytokines and chemokines differentially regulate innate immune cell trafficking during post kala-azar dermal leishmaniasis. J Cell Biochem. (2018) 119:7406–18. doi: 10.1002/jcb.v119.9 29775225

[89] Dayakar A Chandrasekaran S Kuchipudi SV Kalangi SK. Cytokines: key determinants of resistance or disease progression in visceral leishmaniasis: opportunities for novel diagnostics and immunotherapy. Front Immunol. (2019) 10:1–23. doi: 10.3389/fimmu.2019.00670 31024534 PMC6459942

[90] Fievez A Silva-Freitas M Sousa A Santos-Oliveira J Da-Cruz A. Lower levels of leptin are associated with severity parameters in visceral leishmaniasis patients. PloS One. (2019) 14:1–15. doi: 10.1371/journal.pone.0214413 PMC6435192 30913261

[91] Guedes DL da Silva ED Castro MCAB Barbosa Júnior WL Ibarra-Meneses AV Tsoumanis A. Comparison of serum cytokine levels in symptomatic and asymptomatic HIV-*Leishmania* coinfected individuals from a Brazilian visceral leishmaniasis endemic area. PloS Negl Trop Dis. (2022) 16:1–12. doi: 10.1371/journal.pntd.0010542 PMC9246190 35714136

[92] Brenchley JM Price DA Schacker TW Asher TE Silvestri G Rao S. Microbial translocation is a cause of systemic immune activation in chronic HIV infection. Nat Med. (2006) 12:1365–71. doi: 10.1038/nm1511 17115046

[93] Zevin AS McKinnon L Burgener A Klatt NR. Microbial translocation and microbiome dysbiosis in HIV-associated immune activation. Curr Opin HIV AIDS. (2016) 11:182–90. doi: 10.1097/COH.0000000000000234 PMC4752849 26679414

[94] Zicari S Sessa L Cotugno N Ruggiero A Morrocchi E Concato C. Immune activation, inflammation, and non-AIDS co-morbidities in HIV-infected patients under long-term ART. Viruses. (2019) 11:1–19. doi: 10.3390/v11030200 PMC6466530 30818749

[95] Bandera A De Benedetto I Bozzi G Gori A. Altered gut microbiome composition in HIV infection: causes, effects and potential intervention. Curr Opin HIV AIDS. (2018) 13:73–80. doi: 10.1097/COH.0000000000000429 29045252

[96] Hensley-McBain T Berard AR Manuzak JA Miller CJ Zevin AS Polacino P. Intestinal damage precedes mucosal immune dysfunction in SIV infection. Mucosal Immunol. (2018) 11:1429–40. doi: 10.1038/s41385-018-0032-5 PMC6162106 29907866

[97] Younas M Psomas C Reynes C Cezar R Kundura L Portales P. Microbial translocation is linked to a specific immune activation profile in HIV-infected adults with suppressed viremia. Front Immunol. (2019) 10:1–8. doi: 10.3389/fimmu.2019.02185 31572392 PMC6753629

[98] Crakes KR Jiang G. Gut microbiome alterations during HIV/SIV infection: implications for HIV cure. Front Microbiol. (2019) 10:1–10. doi: 10.3389/fmicb.2019.01104 31191468 PMC6539195

[99] O’Connor MA Munson PV Tunggal HC Hajari N Lewis TB Bratt D. Mucosal T helper 17 and T regulatory cell homeostasis correlate with acute simian immunodeficiency virus viremia and responsiveness to antiretroviral therapy in macaques. AIDS Res Hum Retroviruses. (2019) 35:295–305. doi: 10.1089/aid.2018.0184 30398361 PMC6434588

[100] Brenchley JM Douek DC. The mucosal barrier and immune activation in HIV pathogenesis. Curr Opin HIV AIDS. (2008) 3:356–61. doi: 10.1097/COH.0b013e3282f9ae9c PMC2789390 19372990

[101] Marchetti G Tincati C Silvestri G. Microbial translocation in the pathogenesis of HIV infection and AIDS. Clin Microbiol Rev. (2013) 26:2–18. doi: 10.1128/CMR.00050-12 23297256 PMC3553668

[102] Tincati C Douek DC Marchetti G. Gut barrier structure, mucosal immunity and intestinal microbiota in the pathogenesis and treatment of HIV infection. AIDS Res Ther. (2016) 13:1–11. doi: 10.1186/s12981-016-0103-1 27073405 PMC4828806

[103] Pelsers MM Namiot Z Kisielewski W Namiot A Januszkiewicz M Hermens WT. Intestinal-type and liver-type fatty acid-binding protein in the intestine. Tissue distribution and clinical utility. Clin Biochem. (2003) 36:529–35. doi: 10.1016/S0009-9120(03)00096-1 14563446

[104] Hunt PW Brenchley J Sinclair E McCune JM Roland M Page-Shafer K. Relationship between T cell activation and CD4+ T cell count in HIV-seropositive individuals with undetectable plasma HIV RNA levels in the absence of therapy. J Infect Dis. (2008) 197:126–33. doi: 10.1086/524143 PMC3466592 18171295

[105] Xiao Q Yu F Yan L Zhao H Zhang F. Alterations in circulating markers in HIV/AIDS patients with poor immune reconstitution: novel insights from microbial translocation and innate immunity. Front Immunol. (2022) 13:1–14. doi: 10.3389/fimmu.2022.1026070 PMC9618587 36325329

[106] Gioannini TL Weiss JP. Regulation of interactions of gram-negative bacterial endotoxins with mammalian cells. Immunol Res. (2007) 39:249–60. doi: 10.1007/s12026-007-0069-0 17917069

[107] Mudd JC Brenchley JM. Gut mucosal barrier dysfunction, microbial dysbiosis, and their role in HIV disease progression. J Infect Dis. (2016) 214:S58–66. doi: 10.1093/infdis/jiw258 PMC5021240 27625432

[108] Muigai R Shaunak S Gatei DG Wozniak A Bryceson ADM. Jejunal function and pathology in visceral leishmaniasis. Lancet. (1983) 322:476–9. doi: 10.1016/S0140-6736(83)90510-X 6136644

[109] Luz KG Tuon FF Duarte MIS Maia GM Matos P Ramos AMO. Cytokine expression in the duodenal mucosa of patients with visceral leishmaniasis. Rev Soc Bras Med Trop. (2010) 43:393–5. doi: 10.1590/S0037-86822010000400011 20802937

[110] Lewis MD Paun A Romano A Langston H Langner CA Moore IN. Fatal progression of experimental visceral leishmaniasis is associated with intestinal parasitism and secondary infection by commensal bacteria, and is delayed by antibiotic prophylaxis. PloS Pathog. (2020) 16:1–23. doi: 10.1371/journal.ppat.1008456 PMC7179947 32282850

[111] Cappato M. Avaliação do fenômeno da translocação microbianae seus efeitos no agravamento da infecção experimental por Leishmania (Leishmania) infantum em golden hamster. Rio de Janeiro (RJ: Instituto Oswaldo Cruz, Fundação Oswaldo Cruz (2017).

[112] Santos-Oliveira JR Da-Cruz AM. Lipopolysaccharide-induced cellular activation may participate in the immunopathogenesis of visceral leishmaniasis alone or in HIV coinfection. Int J Microbiol. (2012) 2012:1–4. doi: 10.1155/2012/364534 PMC3432364 22956960

[113] Pawelec G Gupta S. Editorial: immunology of aging. Front Immunol. (2019) 10:1–3. doi: 10.3389/fimmu.2019.01614 31354744 PMC6636427

[114] Appay V Sauce D. Assessing immune aging in HIV-infected patients. Virulence. (2017) 8:529–38. doi: 10.1080/21505594.2016.1195536 PMC5538339 27310730

[115] Appay V Almeida JR Sauce D Autran B Papagno L. Accelerated immune senescence and HIV infection. Exp Gerontol. (2007) 42:432–7. doi: 10.1016/j.exger.2006.12.003 17307327

[116] Appay V Sauce D. Immune activation and inflammation in HIV infection: causes and consequences. J Pathol. (2008) 214:231–41. doi: 10.1002/path.v214:2 18161758

[117] Deeks SG Tracy R Douek DC. Systemic effects of inflammation on health during chronic HIV infection. Immunity. (2013) 39:633–45. doi: 10.1016/j.immuni.2013.10.001 PMC4012895 24138880

[118] Xu W Larbi A. Markers of T cell senescence in humans. Int J Mol Sci. (2017) 18:1–13. doi: 10.3390/ijms18081742 PMC5578132 28796199

[119] Brenchley JM Karandikar NJ Betts MR Ambrozak DR Hill BJ Crotty LE. Expression of CD57 defines replicative senescence and antigen-induced apoptotic death of CD8+ T cells. Blood. (2003) 101:2711–20. doi: 10.1182/blood-2002-07-2103 12433688

[120] Henriquez S Lécuroux C Bitu M Avettand-Fenoel V Churaqui F Catalan P. The proportion of CD57+ Cells among effector CD8+ T cells is lower in HIV controllers compared with antiretroviral therapy-treated patients. AIDS. (2019) 33:2137–47. doi: 10.1097/QAD.0000000000002342 31688039

[121] Palmer BE Blyveis N Fontenot AP Wilson CC. Functional and phenotypic characterization of CD57+CD4+ T cells and their association with HIV-induced T cell dysfunction. J Immunol. (2005) 175:8415–23. doi: 10.4049/jimmunol.175.12.8415 16339584

[122] Hove-Skovsgaard M Zhao Y Tingstedt JL Hartling HJ Thudium RF Benfield T. Impact of age and HIV status on immune activation, senescence and apoptosis. Front Immunol. (2020) 11:1–10. doi: 10.3389/fimmu.2020.583569 33117394 PMC7561401

[123] Domínguez-Rodríguez S Tagarro A Foster C Palma P Cotugno N Zicari S. Clinical, virological and immunological subphenotypes in a cohort of early treated HIV-infected children. Front Immunol. (2022) 13:1–12. doi: 10.3389/fimmu.2022.875692 PMC9111748 35592310

[124] Guo XY Qu MM Wang X Wang ZR Song JW Yang BP. Characteristics of blood immune cell profile and their correlation with disease progression in patients infected with HIV. BMC Infect Dis. (2023) 23:1–16. doi: 10.1186/s12879-023-08847-z 38124099 PMC10731693

[125] Elias Junior E Gubert VT Bonin-Jacob CM Puga MAM Gouveia CG Sichinel AH. CD57 T cells associated with immunosenescence in adults living with HIV or AIDS. Immunology. (2024) 171:146–53. doi: 10.1111/imm.v171.1 37880915

[126] Gautam S Kumar R Singh N Singh AK Rai M Sacks D. CD8 T cell exhaustion in human visceral leishmaniasis. J Infect Dis. (2014) 209:290–9. doi: 10.1093/infdis/jit401 PMC3873784 23922369

[127] Akbar AN Henson SM. Are senescence and exhaustion intertwined or unrelated processes that compromise immunity? Nat Rev Immunol. (2011) 11:289–95. doi: 10.1038/nri2959 21436838

[128] Yasuma-Mitobe K Matsuoka M. The roles of coinhibitory receptors in pathogenesis of human retroviral infections. Front Immunol. (2018) 9:1–8. doi: 10.3389/fimmu.2018.02755 30538707 PMC6277675

[129] Lorvik KB Meyer-Myklestad MH Kushekar K Handeland C Medhus AW Lund-Iversen M. Enhanced gut-homing dynamics and pronounced exhaustion of mucosal and blood CD4+ T cells in HIV-infected immunological non-responders. Front Immunol. (2021) 12:1–12. doi: 10.3389/fimmu.2021.744155 PMC8529151 34691047

[130] Vos WAJW Navas A Meeder EMG Blaauw MJT Groenendijk AL van Eekeren LE. HIV immunological non-responders are characterized by extensive immunosenescence and impaired lymphocyte cytokine production capacity. Front Immunol. (2024) 15:1–13. doi: 10.3389/fimmu.2024.1350065 PMC11109418 38779686

[131] Chiku VM Silva KLO de Almeida BFM Venturin GL Leal AAC de Martini CC. PD-1 function in apoptosis of T lymphocytes in canine visceral leishmaniasis. Immunobiology. (2016) 221:879–88. doi: 10.1016/j.imbio.2016.03.007 27016050

[132] Oliveira Silva KL Marin Chiku V Luvizotto Venturin G Correa Leal AA de Almeida BF De Rezende Eugenio F. PD-1 and PD-L1 regulate cellular immunity in canine visceral leishmaniasis. Comp Immunol Microbiol Infect Dis. (2019) 62:76–87. doi: 10.1016/j.cimid.2018.12.002 30711051

[133] Habib S El Andaloussi A Elmasry K Handoussa A Azab M Elsawey A. PDL-1 blockade prevents T cell exhaustion, inhibits autophagy, and promotes clearance of leishmania donovani. Infect Immun. (2018) 86:1–14. doi: 10.1128/IAI.00019-18 PMC5964517 29610255

[134] Esch KJ Juelsgaard R Martinez PA Jones DE Petersen CA. Programmed death 1–mediated T cell exhaustion during visceral leishmaniasis impairs phagocyte function. J Immunol. (2013) 191:5542–50. doi: 10.4049/jimmunol.1301810 PMC3896087 24154626

[135] Grabmeier-Pfistershammer K Stecher C Zettl M Rosskopf S Rieger A Zlabinger GJ. Antibodies targeting BTLA or TIM-3 enhance HIV specific T cell responses in combination with PD-1 blockade. Clin Immunol. (2017) 183:167–73. doi: 10.1016/j.clim.2017.09.002 28882621

[136] de Vrij N Pollmann J Rezende AM Ibarra-Meneses AV Pham TT Hailemichael W. Persistent T cell unresponsiveness associated with chronic visceral leishmaniasis in HIV-coinfected patients. Commun Biol. (2024) 7:1–13. doi: 10.1038/s42003-024-06225-2 38702419 PMC11068874

[137] Papagno L Spina CA Marchant A Salio M Rufer N Little S. Immune activation and CD8+ T-cell differentiation towards senescence in HIV infection. PloS Biol. (2004) 2:173–85. doi: 10.1371/journal.pbio.0020020 PMC340937 14966528

[138] Chauvin M Sauce D. Mechanisms of immune aging in HIV. Clin Sci. (2022) 136:61–80. doi: 10.1042/CS20210344 34985109

[139] Douek D McFarland RD Keiser PH Gage EA Massey JM Haynes BF. Changes in thymic function with age and during the treatment of HIV infection. Nature. (1998) 396:690–5. doi: 10.1038/25374 9872319

[140] Molina-Pinelo S Vallejo A Díaz L Soriano-Sarabia N Ferrando-Martínez S Resino S. Premature immunosenescence in HIV-infected patients on highly active antiretroviral therapy with low-level CD4 T cell repopulation. J Antimicrob Chemother. (2009) 64:579–88. doi: 10.1093/jac/dkp248 19608579

[141] Quiros-Roldan E Serana F Chiarini M Zanotti C Sottini A Gotti D. Effects of combined antiretroviral therapy on B- and T-cell release from production sites in long-term treated HIV+ patients. J Transl Med. (2012) 10:1–11. doi: 10.1186/1479-5876-10-94 22591651 PMC3481359

[142] Dalzini A Ballin G Dominguez-Rodriguez S Rojo P Petrara MR Foster C. Size of HIV reservoir is associated with telomere shortening and immunosenescence in early-treated european children with perinatally acquired HIV. J Int AIDS Soc. (2021) 24:1–10. doi: 10.1002/jia2.v24.11 PMC8604380 34797948

[143] Ruiz Pérez M Vandenabeele P Tougaard P. The thymus road to a T cell: migration, selection, and atrophy. Front Immunol. (2024) 15:1443910. doi: 10.3389/fimmu.2024.1443910 39257583 PMC11384998

[144] Kolte L. Thymic function in HIV-infection. Dan Med J. (2013) 60:1–24. 23651726

[145] Ye P Kirschner D Kourtis A. The thymus during HIV disease: role in pathogenesis and in immune recovery. Curr HIV Res. (2004) 2:177–83. doi: 10.2174/1570162043484898 15078181

[146] Young CD Angel JB. HIV infection of thymocytes inhibits IL-7 activity without altering CD127 expression. Retrovirology. (2011) 8:1–6. doi: 10.1186/1742-4690-8-72 21920046 PMC3182983

[147] Fiume G Scialdone A Albano F Rossi A Tuccillo FM Rea D. Impairment of T cell development and acute inflammatory response in HIV tat transgenic mice. Sci Rep. (2015) 5:1–14. doi: 10.1038/srep13864 PMC4561375 26343909

[148] Furler RL Newcombe KL Del Rio Estrada PM Reyes-Terán G Uittenbogaart CH Nixon DF. Histoarchitectural deterioration of lymphoid tissues in HIV infection and in aging. AIDS Res Hum Retroviruses. (2019) 35:1148–59. doi: 10.1089/aid.2019.0156 PMC6862967 31474115

[149] Lu IN Ahmad F Jacobs R Schmidt RE Meyer-Olson D. Optimal gating strategy for determining CD4+ Recent thymic emigrants in human immunodeficiency virus-1 infected patients. Viral Immunol. (2014) 27:179–84. doi: 10.1089/vim.2013.0132 24766582

[150] Douek DC Koup RA McFarland RD Sullivan JL Luzuriaga K. Effect of HIV on thymic function before and after antiretroviral therapy in children. J Infect Dis. (2000) 181:1479–82. doi: 10.1086/jid.2000.181.issue-4 10762580

[151] Hatzakis A Touloumi G Karanicolas R Karafoulidou A Mandalaki T Anastassopoulou C. Effect of recent thymic emigrants on progression of HIV disease. Lancet. (2000) 355:599–604. doi: 10.1016/S0140-6736(99)10311-8 10696979

[152] Sodora DL Milush JM Ware F Wozniakowski A Montgomery L McClure HM. Decreased levels of recent thymic emigrants in peripheral blood of simian immunodeficiency virus-infected macaques correlate with alterations within the thymus. J Virol. (2002) 76:9981–90. doi: 10.1128/JVI.76.19.9981-9990.2002 PMC136511 12208974

[153] Gaardbo JC Hartling HJ Ronit A Thorsteinsson K Madsen HO Springborg K. Different immunological phenotypes associated with preserved CD4+ T cell counts in HIV-infected controllers and viremic long term non-progressors. PloS One. (2013) 8:1–10. doi: 10.1371/journal.pone.0063744 PMC3655944 23696852

[154] Ferrando-Martinez S De Pablo-Bernal RS De Luna-Romero M De Ory SJ Genebat M Pacheco YM. Thymic function failure is associated with human immunodeficiency virus disease progression. Clin Infect Dis. (2017) 64:1191–7. doi: 10.1093/cid/cix095 PMC6248450 28158588

[155] Rb-silva R Nobrega C Azevedo C Athayde E. Thymic function as a predictor of immune recovery in chronically HIV-infected patients initiating antiretroviral therapy. Front Immunol. (2019) 10:1–13. doi: 10.3389/fimmu.2019.00025 30804925 PMC6370619

[156] Briceño O Chávez-Torres M Peralta-Prado A Garrido-Rodríguez D Romero-Mora K Pinto-Cardoso S. Associations between recent thymic emigrants and CD4+ T-cell recovery after short-term antiretroviral therapy initiation. AIDS. (2020) 34:501–11. doi: 10.1097/QAD.0000000000002458 PMC7050791 31794524

[157] Guedes MCS Carvalho-Silva WHV Andrade-Santos JL Brelaz-de-Castro MCA Souto FO Montenegro LML. HIV-induced thymic insufficiency and aging-related immunosenescence on immune reconstitution in ART-treated patients. Vaccines (Basel). (2024) 12:1–7. doi: 10.3390/vaccines12060612 PMC11209262 38932341

[158] Fabre-Mersseman V Dutrieux J Louise A Rozlan S Lamine A Parker R. CD4+ Recent thymic emigrants are infected by HIV *in vivo*, implication for pathogenesis. AIDS. (2011) 25:1153–62. doi: 10.1097/QAD.0b013e3283471e89 21505308

[159] Losada-Barragán M Umanã-Pérez A Cuervo-Escobar S Berbert LR Porrozzi R Morgado FN. Protein malnutrition promotes dysregulation of molecules involved in T cell migration in the thymus of mice infected with leishmania infantum. Sci Rep. (2017) 7:1–13. doi: 10.1038/srep45991 28397794 PMC5387407

[160] Brazin KN Mallis RJ Das DK Feng Y Hwang W Wang JH. Structural features of the AβTCR mechanotransduction apparatus that promote pMHC discrimination. Front Immunol. (2015) 6:1–13. doi: 10.3389/fimmu.2015.00441 26388869 PMC4558533

[161] Salameire D Solly F Fabre B Lefebvre C Chauvet M Gressin R. Accurate detection of the tumor clone in peripheral T-cell lymphoma biopsies by flow cytometric analysis of TCR-VB Repertoire. Mod Pathol. (2012) 25:1246–57. doi: 10.1038/modpathol.2012.74 22627740

[162] Yan L Wang Z Cui C Guan X Dong B Zhao M. Comprehensive immune characterization and T-cell receptor repertoire heterogeneity of retroperitoneal liposarcoma. Cancer Sci. (2019) 110:3038–48. doi: 10.1111/cas.v110.10 PMC6778648 31385405

[163] Costa RP Chaves ACL Ludwig I Paulo Ä. T-cell repertoire analysis in acute and chronic human chagas’ Disease: differential frequencies of vbeta5 expressing T cells. Scand J Immunol. (2000) 51:511–9. doi: 10.1046/j.1365-3083.2000.00706.x 10792844

[164] Fernández-Mestre M Jaraquemada D Bruno RE Caro J Layrisse Z. Analysis of the T-cell receptor beta-chain variable-region (Vbeta) repertoire in chronic human Chagas’ disease. Tissue Antigens. (2002) 60:10–5. doi: 10.1034/j.1399-0039.2002.600102.x 12366778

[165] Giacoia-Gripp CBW Neves I Galhardo MC Morgado MG. Flow cytometry evaluation of the T-cell receptor Vβ Repertoire among HIV infected individuals before and after antiretroviral therapy. J Clin Immunol. (2005) 25:116–26. doi: 10.1007/s10875-005-2817-z 15821888

[166] Yin L Kou ZC Rodriguez C Hou W Goodenow MM Sleasman JW. Antiretroviral therapy restores diversity in the T-cell receptor V repertoire of CD4 T-cell subpopulations among human immunodeficiency virus type 1-infected children and adolescents. Clin Vaccine Immunol. (2009) 16:1293–301. doi: 10.1128/CVI.00074-09 PMC2745006 19605599

[167] Hernández DM Valderrama S Gualtero S Hernández C Quijano S. Loss of T-cell multifunctionality and TCR-V β Repertoire against Epstein-Barr Virus is associated with worse prognosis and clinical parameters in HIV+ patients. Front Immunol. (2018) 9:1–15. doi: 10.3389/fimmu.2018.02291 30337929 PMC6180205

[168] Clarêncio J de Oliveira CI Bomfim G Pompeu MM Teixeira MJ Barbosa TC. Characterization of the T-cell receptor Vbeta repertoire in the human immune response against Leishmania parasites. Infect Immun. (2006) 74:4757–65. doi: 10.1128/IAI.00265-06 PMC1539606 16861664

[169] Ferraz R Cunha CF Pimentel MI Lyra MR Schubach AO Mendonça SCF. T-cell receptor Vβ repertoire of CD8+ T-lymphocyte subpopulations in cutaneous leishmaniasis patients from the state of Rio de Janeiro, Brazil. Mem Inst Oswaldo Cruz. (2015) 110:596–605. doi: 10.1590/0074-02760150039 26107186 PMC4569821

[170] Kariminia A Bourreau E Ronet C Couppié P Sainte-Marie D Tacchini-Cottier F. Selective expression of the V beta 14 T cell receptor on *Leishmania guyanensis*-specific CD8+ T cells during human infection. J Infect Dis. (2007) 195:739–47. doi: 10.1086/510912 17262718

[171] Cota G de Sousa MR Assis TSM Pinto BF Rabello A. Exploring prognosis in chronic relapsing visceral leishmaniasis among HIV-infected patients: circulating leishmania DNA. Acta Trop. (2017) 172:186–91. doi: 10.1016/j.actatropica.2017.05.011 28501450

[172] Clemente W Vidal E Girão E Ramos ASD Govedic F Merino E. Risk factors, clinical features and outcomes of visceral leishmaniasis in solid-organ transplant recipients: A retrospective multicenter case–control study. Clin Microbiol Infect. (2015) 21:89–95. doi: 10.1016/j.cmi.2014.09.002 25636932

[173] Simão JC Victória C Fortaleza CMCB. Predictors of relapse of visceral leishmaniasis in inner São Paulo state, Brazil. Int J Infect Dis. (2020) 95:44–9. doi: 10.1016/j.ijid.2020.02.028 32088340

[174] Horrillo L Castro A Matía B Molina L Martínez JG Jaqueti J. Clinical aspects of visceral leishmaniasis caused by *L. Infantum* in adults. Ten years of experience of the largest outbreak in europe: what have we learned? Parasit Vectors. (2019) 12:1–11. doi: 10.1186/s13071-019-3628-z 31340851 PMC6657057

[175] Abongomera C Diro E Vogt F Tsoumanis A Mekonnen Z Admassu H. The risk and predictors of visceral leishmaniasis relapse in human immunodeficiency virus-coinfected patients in Ethiopia: A retrospective cohort study. Clin Infect Dis. (2017) 65:1703–10. doi: 10.1093/cid/cix607 PMC5848226 29020196

[176] Verrest L Kip AE Musa AM Schoone GJ Schallig HDFH Mbui J. Blood parasite load as an early marker to predict treatment response in visceral leishmaniasis in eastern africa. Clin Infect Dis. (2021) 73:775–82. doi: 10.1093/cid/ciab124 PMC8423463 33580234

[177] Burza S Sinha PK Mahajan R Lima MA Mitra G Verma N. Risk factors for visceral leishmaniasis relapse in immunocompetent patients following treatment with 20 mg/kg liposomal amphotericin B (Ambisome) in bihar, India. PloS Negl Trop Dis. (2014) 8:1–8. doi: 10.1371/journal.pntd.0002536 PMC3879206 24392166

[178] Berenguer J Cosín J Miralles P López JC Padilla B. Discontinuation of secondary anti-*Leishmania* prophylaxis in HIV-infected patients who have responded to highly active antiretroviral therapy. AIDS. (2000) 14:2946–8. doi: 10.1097/00002030-200012220-00020 11153679

[179] Casado JL Lopez-Velez R Pintado V Quereda C Antela A Moreno S. Relapsing visceral leishmaniasis in HIV-infected patients undergoing successful protease inhibitor therapy. Eur J Clin Microbiol Infect Dis. (2001) 20:202–5. doi: 10.1007/s100960100457 11347673

[180] Bossolasco S Gaiera G Olchini D Gulletta M Martello L Bestetti A. Real-time PCR assay for clinical management of human immunodeficiency virus-infected patients with visceral leishmaniasis [published correction appears in J Clin Microbiol. 2004 Apr;42(4):1858. J Clin Microbiol. (2003) 41:5080–4. doi: 10.1128/JCM.41.11.5080-5084.2003 PMC262523 14605142

[181] Mira JA Corzo JE Rivero A Macias J De Leon FL Torre-Cisneros J. Frequency of visceral leishmaniasis relapses in human immunodeficiency virus-infected patients receiving highly active antiretroviral therapy. Am J Trop Med Hyg. (2004) 70:298–301. doi: 10.4269/ajtmh.2004.70.298 15031520

[182] Molina I Falcó V Crespo M Riera C Ribera E Curran A. Efficacy of liposomal amphotericin B for secondary prophylaxis of visceral leishmaniasis in HIV-infected patients. J Antimicrob Chemother. (2007) 60:837–42. doi: 10.1093/jac/dkm294 17684055

[183] Bourgeois N Lachaud L Reynes J Rouanet I Mahamat A Bastien P. Long-term monitoring of visceral leishmaniasis in patients with AIDS: relapse risk factors, value of polymerase chain reaction, and potential impact on secondary prophylaxis. J Acquir Immune Defic Syndr. (2008) 48:13–9. doi: 10.1097/QAI.0b013e318166af5d 18300698

[184] ter Horst R Collin SM Ritmeijer K Bogale A Davidson RN. Concordant HIV infection and visceral leishmaniasis in Ethiopia: the influence of antiretroviral treatment and other factors on outcome. Clin Infect Dis. (2008) 46:1702–9. doi: 10.1086/587899 18419422

[185] Bhattacharyya T Ayandeh A Falconar AK Sundar S El-safi S Gripenberg MA. IgG1 as a potential biomarker of post-chemotherapeutic relapse in visceral leishmaniasis, and adaptation to a rapid diagnostic test. PloS Negl Trop Dis. (2014) 8:1–11. doi: 10.1371/journal.pntd.0003273 PMC4207679 25340782

[186] Marlais T Bhattacharyya T Singh OP Mertens P Gilleman Q Thunissen C. Visceral Leishmaniasis IgG1 Rapid Monitoring of Cure vs. Relapse, and Potential for Diagnosis of Post Kala-Azar Dermal Leishmaniasis. Front Cell Infect Microbiol. (2018) 8:1–10. doi: 10.3389/fcimb.2018.00427 30619774 PMC6300496

[187] Mollett G Bremer Hinckel BC Bhattacharyya T Marlais T Singh OP Mertens P. Detection of immunoglobulin G1 against RK39 improves monitoring of treatment outcomes in visceral leishmaniasis. Clin Infect Dis. (2019) 13:1130–5. doi: 10.1093/cid/ciy1062 PMC6743847 30541022

[188] Mondal D Ghosh P Chowdhury R Halleux C Ruiz-postigo JA Alim A. Relationship of serum antileishmanial antibody with development of visceral dermal leishmaniasis and visceral leishmaniasis relapse. Front Microbiol. (2019) 10:1–12. doi: 10.3389/fmicb.2019.02268 31649631 PMC6795025

